# Scheduled intravenous acetaminophen *versus* thoracic epidural analgesia for postoperative pain control after minimally invasive gastrectomy: multicentre randomized non-inferiority trial

**DOI:** 10.1093/bjsopen/zrag031

**Published:** 2026-04-27

**Authors:** Jun Kinoshita, Saki Hayashi, Toshikatsu Tsuji, Hideki Moriyama, Yuki Yamazaki, Yuto Kitano, Tomoya Tsukada, Masahide Kaji, Takahisa Yamaguchi, Shinichi Kadoya, Yasumichi Yagi, Masanari Shimada, Shingo Soga, Sachio Fushida, Takumi Taniguchi, Noriyuki Inaki

**Affiliations:** Department of Gastrointestinal Surgery, Kanazawa University Hospital, Kanazawa, Japan; Department of Gastrointestinal Surgery, Kanazawa University Hospital, Kanazawa, Japan; Department of Gastrointestinal Surgery, Kanazawa University Hospital, Kanazawa, Japan; Department of Gastrointestinal Surgery, Kanazawa University Hospital, Kanazawa, Japan; Department of Surgery, Public Central Hospital of Matto Ishikawa, Hakusan, Japan; Department of Surgery, Toyama Red Cross Hospital, Toyama, Japan; Department of Surgery, Toyama Prefectural Central Hospital, Toyama, Japan; Department of Surgery, Toyama Prefectural Central Hospital, Toyama, Japan; Department of Surgery, Ishikawa Prefectural Central Hospital, Kanazawa, Japan; Department of Surgery, Ishikawa Prefectural Central Hospital, Kanazawa, Japan; Department of Surgery, Kanazawa Medical Center, Kanazawa, Japan; Department of Surgery, Fukui-ken Saiseikai Hospital, Fukui, Japan; Department of Surgery, JCHO Kanazawa Hospital, Kanazawa, Japan; Department of Gastrointestinal Surgery, Kanazawa University Hospital, Kanazawa, Japan; Non-Profit Organization (NPO) Digestive Disease Support Organization Study Group, Kanazawa, Japan; Department of Anesthesiology and Intensive Care Medicine, Kanazawa University Hospital, Kanazawa, Japan; Department of Gastrointestinal Surgery, Kanazawa University Hospital, Kanazawa, Japan

**Keywords:** enhanced recovery after surgery (ERAS), multimodal analgesia, opioid-sparing, perioperative management

## Abstract

**Background:**

Thoracic epidural analgesia (TEA) is widely used for postoperative pain control in abdominal surgery, but its role in minimally invasive procedures remains unclear. This pragmatic multicentre open-label randomized non-inferiority trial evaluated whether scheduled intravenous acetaminophen combined with local wound infiltration provides non-inferior analgesia to TEA following minimally invasive gastrectomy.

**Methods:**

Adults with gastric adenocarcinoma categorized as clinical stage I–III (according to the Japanese classification of gastric carcinoma) who were undergoing laparoscopic or robot-assisted gastrectomy were randomized 1 : 1 to TEA or scheduled intravenous acetaminophen (1 g every 6 hours (h) for 72 h). All patients received wound infiltration with ropivacaine. Participants and clinicians were not blinded to the randomization; statisticians were masked. The primary endpoint in the study was the proportion of patients with a numerical rating scale (NRS) pain score ≥ 4 at rest 24 h after surgery, with a prespecified non-inferiority margin of 20 percentage points. Analyses used the modified intention-to-treat population.

**Results:**

Between June 2020 and May 2024, 140 patients were randomized and 135 were analysed (TEA 68, acetaminophen 67). At 24 h after surgery, 27.9% of patients in the TEA group and 23.9% in the acetaminophen group had an NRS score ≥ 4 at rest (risk difference −4.1%; 95% confidence interval −24.3 to 16.6), confirming non-inferiority. Pain trajectories, rescue analgesic use, patient satisfaction, and recovery were comparable between the two groups. No patient required rescue opioids, and there were no serious catheter- or acetaminophen-related adverse events.

**Conclusion:**

Scheduled intravenous acetaminophen with wound infiltration was non-inferior to TEA for postoperative pain after minimally invasive gastrectomy and provided safe, effective, and opioid-sparing analgesia consistent with enhanced recovery principles. Registration number: UMIN000039505 (https://www.umin.ac.jp/english/).

## Teaser

Thoracic epidural analgesia (TEA) is commonly used after abdominal surgery, but its necessity in minimally invasive gastrectomy remains uncertain. In this pragmatic multicentre randomized non-inferiority trial, scheduled intravenous acetaminophen combined with wound infiltration achieved non-inferior postoperative pain control compared with TEA, with comparable pain trajectories and recovery, and no increase in adverse events. These findings suggest scheduled intravenous acetaminophen is a safe, effective, and opioid-sparing alternative to TEA, supporting simplified analgesic strategies aligned with enhanced recovery after minimally invasive gastrectomy.

## Introduction

Optimized postoperative analgesia is a cornerstone of enhanced recovery after surgery (ERAS) protocols, emphasizing the importance of multimodal analgesia, with acetaminophen as a key component^1,2^. In a previous randomized clinical trial (RCT) involving patients undergoing gastrectomy for gastric cancer, scheduled intravenous acetaminophen in combination with thoracic epidural analgesia (TEA) significantly reduced postoperative pain compared with TEA alone^3^. However, subgroup analyses showed that this benefit was more pronounced after open gastrectomy than after laparoscopic procedures, where the additive effect of acetaminophen appeared limited^3^.

TEA has long been considered the gold standard for major abdominal surgery because of its potent analgesic effect^4^. Nevertheless, its technical success rate remains variable (70–80%), and it carries well recognized risks, including hypotension, urinary retention, and, rarely, catastrophic neuraxial complications^5,6^. With the increasing adoption of minimally invasive surgery, the need for and overall benefit of routine TEA have been called into question. Indeed, the 2019 update of the ERAS^®^ Society guidelines^7^ for colorectal surgery no longer recommend routine TEA for laparoscopic procedures, reflecting a shift towards less invasive pain management.

Minimally invasive gastrectomy has become an established global standard with proven safety and oncological feasibility^8–10^. Although one RCT comparing TEA with intravenous opioid analgesia after laparoscopic gastrectomy reported faster bowel recovery and slightly lower early pain scores with TEA, it showed no improvement in overall recovery^11^. However, no study to date has demonstrated the non-inferiority of a non-epidural analgesic regimen compared with TEA in this context. Although current ERAS^®^ Society guidelines for gastrectomy recommend TEA for open procedures^(12)^, supporting evidence for minimally invasive gastrectomy remains limited, and no procedure-specific analgesic protocol has yet been established.

In recent years, opioid-sparing and opioid-free multimodal analgesic strategies have drawn increasing attention, with acetaminophen as a key component^13,14^. These approaches have emerged in response to growing evidence that opioid-based perioperative analgesia offers no clear advantage in pain control compared with multimodal non-opioid regimens, yet is associated with adverse effects and an elevated risk of prolonged use or dependence, factors that have contributed to the global opioid crisis^15,16^.

Accordingly, the present study was designed as a pragmatic multicentre randomized non-inferiority trial to determine whether a scheduled intravenous acetaminophen-based regimen is non-inferior to TEA for postoperative pain control following minimally invasive gastrectomy.

## Methods

### Study design

This multicentre pragmatic randomized open-label non-inferiority trial was conducted by the Gastrointestinal Cancer Conference Study Group in Japan. The study evaluated whether scheduled intravenous acetaminophen is non-inferior to TEA for postoperative pain control following minimally invasive gastrectomy. The protocol was approved by the Institutional Review Board of Kanazawa University (Approval no. 2019-007) and by the ethics committees of all participating centres. The trial followed the Declaration of Helsinki and CONSORT guidelines and was registered with the University Hospital Medical Information Network (UMIN) Clinical Trials Registry (UMIN000039505). The full study protocol is available in the *Supplementary material*.

### Participants

Adults (aged ≥ 20 years) with histologically confirmed gastric adenocarcinoma, categorized as clinical stage I–III according to the Japanese classification of gastric carcinoma (3rd English edition)^17^, who were scheduled to undergo curative laparoscopic or robot-assisted gastrectomy were eligible for inclusion in the study. Key eligibility criteria included an Eastern Cooperative Oncology Group performance status of 0–1 and adequate organ function within the 4 weeks before surgery. Patients were excluded if they had another active malignancy, significant cardiopulmonary co-morbidities, or contraindications to TEA, were undergoing ongoing systemic corticosteroid therapy, were pregnant or breastfeeding, or had known hypersensitivity to acetaminophen, non-steroidal anti-inflammatory drugs, or local anaesthetics. Patients deemed unsuitable by the investigator were also excluded from the study.

### Randomization

After providing written informed consent, patients were randomly assigned in a 1 : 1 ratio to receive either TEA or scheduled intravenous acetaminophen. Randomization was performed centrally using a computer-generated minimization method, with participating institution and type of gastrectomy (distal *versus* proximal/total) specified as minimization factors to ensure balance between groups. To reduce predictability, the minimization algorithm incorporated a random element, such that treatment assignment was not purely deterministic. Allocation was implemented sequentially at the time of patient enrolment and was managed by a data manager independent of recruitment and treatment, thereby ensuring allocation concealment.

Because of the nature of the interventions, neither patients nor clinical staff were blinded; however, statisticians remained blinded to group allocation until database lock.

### Surgical and anaesthetic management

All procedures were performed in accordance with the 5th edition of the Japanese gastric cancer treatment guidelines^18^. Port placement, incision length, and reconstruction method were left to the discretion of the operating surgeon. Because this was a pragmatic multicentre trial, intraoperative anaesthetic management followed each institution's routine practice. To ensure consistency in multimodal analgesia, all patients received wound infiltration with 10 ml of 0.75% ropivacaine diluted with 10 ml saline, which was injected into the subfascial (preperitoneal) space under direct vision at the end of the operation.

### Interventions

In the TEA group, a thoracic epidural catheter (T8–T10) was inserted before induction of anaesthesia. The recommended solution was 0.2% ropivacaine combined with fentanyl (total 500–1000 µg) diluted to 300 ml. Continuous basal infusion with optional patient-controlled epidural analgesia was encouraged, targeting a numerical rating scale (NRS) pain score of < 4. Delivery systems and infusion parameters followed each institution's standard practice but were applied uniformly to all participants within each site. Institution-specific TEA regimens and the number of patients enrolled in each treatment group are summarized in *Table S1*. The epidural catheter was removed on postoperative day (POD) 3.

In the acetaminophen group, scheduled intravenous acetaminophen (Acelio IV; Terumo Corporation, Tokyo, Japan) was administered immediately after surgery (1000 mg for patients weighing ≥ 50 kg or 15 mg/kg for those weighing < 50 kg) and repeated every 6 hours (h) until POD 3.

Rescue analgesics for breakthrough pain were permitted at the discretion of the attending physician, with both the type and frequency of use recorded. In patients receiving TEA, patient-controlled epidural analgesia bolus doses were considered part of the epidural analgesic regimen and were not counted as rescue analgesia. Routine administration of oral or intravenous opioids for rescue analgesia was not allowed.

### Pain assessment and safety monitoring

Pain intensity was assessed using NRS pain scores (0 = no pain, 10 = worst pain imaginable) and recorded three times daily, at rest and during coughing, by trained ward nurses. Analgesia-related adverse events, including sedation, postoperative nausea and vomiting (PONV), and urinary retention, were documented prospectively. PONV was defined as nausea or vomiting requiring at least one additional dose of metoclopramide. Surgical complications were graded according to the Clavien–Dindo classification^19^, and laboratory toxicities were evaluated using Common Terminology Criteria for Adverse Events (CTCAE) v4.0^20^. From POD 4 onward, analgesic management was at the discretion of the attending physician.

### Endpoints

The primary endpoint in this study was the proportion of patients with an NRS score ≥ 4 at rest 24 h after surgery. Secondary endpoints were the area under the NRS–time curve during the first 72 h (AUC_72_); the time course of NRS scores at rest and during coughing; the number of rescue analgesic doses through POD 3; patient satisfaction, assessed using a seven-point Likert scale (scores ranging from 1 to 7, with lower scores indicating better satisfaction); anaesthetic and operative time; postoperative recovery parameters, including time to first flatus, defaecation, ambulation, urinary catheter removal, and length of hospital stay; and safety outcomes, encompassing surgical and analgesia-related complications. Patients were followed until hospital discharge.

Exploratory post hoc analyses included the proportion of patients with NRS score ≥ 4 at rest at 48 and 72 h and during coughing at 24, 48, and 72 h.

### Sample size calculation

Based on a previous multicentre RCT in gastric cancer surgery^3^, 31% of patients receiving TEA after laparoscopic gastrectomy reported an NRS score ≥ 4 at rest 24 h after surgery. Assuming a similar incidence in this trial and setting a non-inferiority margin of 20 percentage points in absolute risk difference (RD), a sample size of 64 patients per group was required to achieve 80% power with a one-sided α of 0.05. Allowing for an anticipated 10% attrition rate, the target enrolment was set at 140 patients (70 per group).

### Statistical analysis

Efficacy analyses were conducted in the modified intention-to-treat (mITT) population, defined as all randomized patients who underwent gastrectomy and received their allocated intervention, excluding those who met predefined discontinuation criteria, such as conversion to open surgery, failed epidural catheter placement, or intraoperative discovery of clinical stage IV disease. A per-protocol analysis, limited to patients who fully adhered to their assigned intervention, was prespecified as a sensitivity analysis.

For the primary endpoint, defined as the proportion of patients with an NRS pain score ≥ 4 at rest 24 h after surgery, the absolute RD (acetaminophen−TEA) and its two-sided 95% confidence interval (c.i.) were estimated using the Newcombe method for differences between two independent proportions. Non-inferiority was concluded if the upper bound of the 95% c.i. was less than the prespecified non-inferiority margin of +20 percentage points. Although the study protocol originally specified the Wald method for the unadjusted c.i., the Newcombe method was used in the final analysis to improve coverage properties. To align the analysis with the one-sided significance level (α = 0.05) used for the sample size calculation, a two-sided 90% c.i. was also calculated and reported as a supportive analysis.

In addition, an adjusted analysis of the primary endpoint was conducted using a multivariable logistic regression model including the prespecified minimization factors (institution and type of gastrectomy), with further adjustment for surgical approach (laparoscopic or robot-assisted). Adjusted RDs were derived from model-based marginal predicted probabilities.

Secondary outcomes were analysed without adjustment for multiplicity, and the results were interpreted as exploratory. Continuous variables were analysed using Student’s *t* test or the Mann–Whitney *U* test, whereas categorical variables were compared using the χ^2^ test or Fisher's exact test, as appropriate. The AUC_72_ was calculated using the linear trapezoidal method. Patient satisfaction was expressed as the proportion of days with a Likert score of ≤ 3 from POD 1 to POD 7 and was summarized as the median with interquartile range (i.q.r.). Detailed statistical methods for secondary outcomes are provided in the *supplementary methods*.

Exploratory post hoc analyses of repeated binary pain outcomes, defined as an NRS score ≥ 4, were performed using logistic generalized linear mixed models (GLMMs). Adjusted RDs between treatment groups at each postoperative time point were calculated from these models. Methodological details are provided in the *supplementary methods*.

Missing data were handled using a complete-case approach.

All statistical analyses were performed using StatFlex version 7.0 (Artech, Osaka, Japan) and R version 4.5.1 (R Foundation for Statistical Computing, Vienna, Austria). Two-sided *P* < 0.05 was considered statistically significant, except for the predefined non-inferiority criterion.

## Results

### Patient disposition and baseline characteristics

**Fig. 1:**
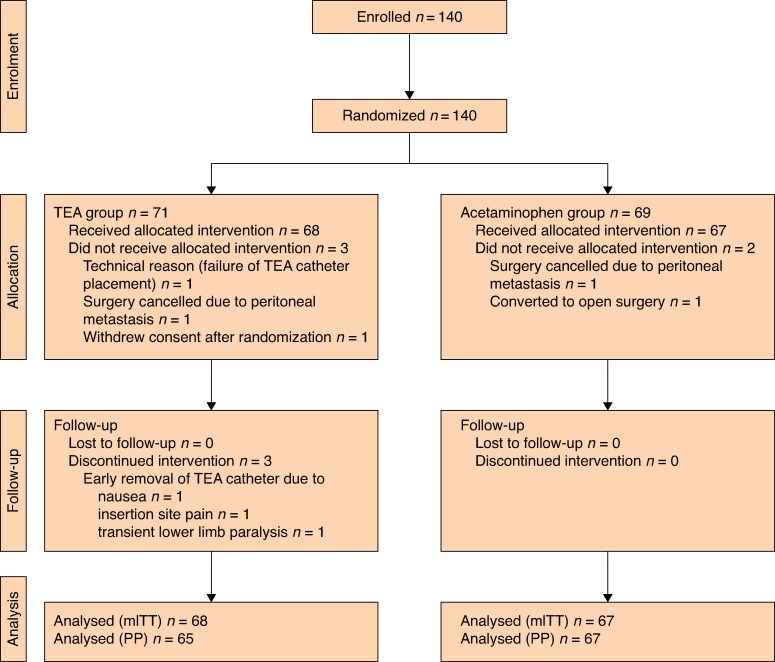
CONSORT flow diagram of patient enrolment and analysis Between June 2020 and May 2024, 140 patients were randomized to TEA (*n* = 71) or scheduled intravenous acetaminophen (*n* = 69). After exclusions based on predefined criteria, 135 patients (TEA, *n* = 68; acetaminophen, *n* = 67) were included in the (mITT) analysis, and 132 were included in the PP analysis. TEA, thoracic epidural analgesia. mITT, modified intention-to-treat; PP, per-protocol.

Between June 2020 and May 2024, 140 patients were enrolled across seven institutions and randomized to either the TEA group (71 patients) or the acetaminophen group (69 patients). After exclusions according to predefined criteria, including conversion to open surgery and withdrawal of consent (*Fig. 1*), 135 patients (68 in the TEA group, 67 in the acetaminophen group) comprised the mITT population, and 132 patients (65 in the TEA group, 67 in the acetaminophen group) comprised the per-protocol population.

Baseline demographics, tumour stage, and surgical procedures were well balanced between the two groups (*Table 1*). For all prespecified study outcomes, including the primary endpoint, complete data were available for patients in the modified intention-to-treat population, except for patient satisfaction, which was evaluable for 58 patients in the TEA group and 59 patients in the acetaminophen group.

**Table 1:** Baseline characteristics of the study participants (modified intention-to-treat population)

	TEA (*n* = 68)	Acetaminophen (*n* = 67)
Age (years), median (i.q.r.)	72.5 (62.5–75.0)	70 (60.5–74.8)
**Sex**		
Male	44 (64.7%)	48 (71.6%)
Female	24 (35.3%)	19 (28.4%)
BMI (kg/m^2^)	23.0 (20.1–24.9)	22.5 (21.1–25.0)
**ASA physical status**		
Grade I	12 (17.6%)	12 (17.9%)
Grade II	48 (70.6%)	50 (74.6%)
Grade III	8 (11.8%)	5 (7.5%)
Clinical stage*		
I	44 (64.7%)	44 (65.7%)
II	13 (19.1%)	13 (19.4%)
III	11 (16.2%)	10 (14.9%)
**Type of gastrectomy**		
Distal	58 (85.3%)	57 (85.0%)
Proximal	3 (4.4%)	5 (7.5%)
Total	7 (10.3%)	5 (7.5%)
**Surgical approach**		
Laparoscopic	34 (50.0%)	32 (47.8%)
Robot-assisted	34 (50.0%)	35(52.2%)
**Lymph node dissection**		
D1	1 (1.5%)	1 (1.5%)
D1+	47 (69.1%)	40 (59.7%)
D2	20 (29.4%)	26 (38.8%)

Values are *n* (%) unless otherwise stated. *Clinical staging was as per the Japanese classification of gastric carcinoma^17^. TEA, thoracic epidural analgesia; i.q.r., interquartile range; BMI, body mass index; ASA, American Society of Anesthesiologists.

### Primary endpoint

**Fig. 2:**
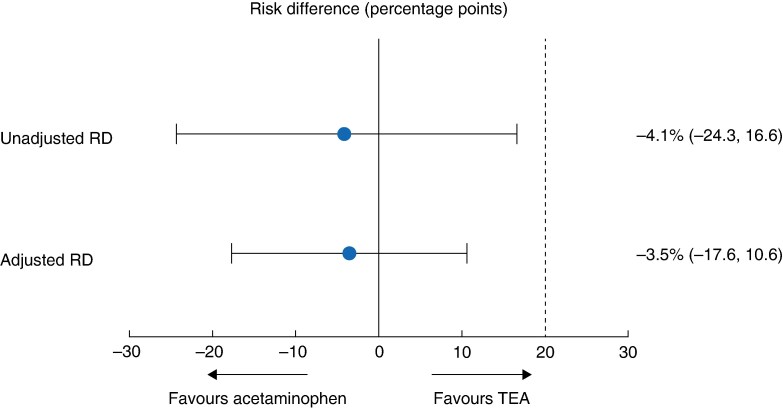
Forest plot of absolute RD (acetaminophen−TEA) for the primary endpoint in the modified intention-to-treat population Values in parentheses are 95% confidence intervals. Unadjusted and adjusted absolute RDs with corresponding 95% confidence intervals are shown for the primary endpoint, defined as the proportion of patients with a numerical rating scale pain score ≥ 4 at rest 24 hours after surgery. Adjusted estimates were derived from a multivariable logistic regression model including institution, type of gastrectomy, and surgical approach as covariates. The vertical dashed line denotes the prespecified non-inferiority margin of +20 percentage points. RD, risk difference; TEA, thoracic epidural analgesia.

In the mITT population, inadequate pain control, defined as an NRS score of ≥ 4 at rest 24 h after surgery, was observed in 27.9% (19 of 68) and 23.9% (16 of 67) of patients in the TEA and acetaminophen groups, respectively (*Fig. 2*). The RD (acetaminophen−TEA) was −4.1% (two-sided 95% c.i. −24.3 to 16.6), and the upper bound of the c.i. did not exceed the non-inferiority margin of +20 percentage points, thereby meeting the prespecified criterion for non-inferiority.

Using a two-sided 90% c.i., corresponding to the one-sided α = 0.05 used for the sample size calculation, the RD was −21.2 to 13.4%, with an unchanged conclusion.

In an adjusted multivariable logistic regression analysis accounting for institution, type of gastrectomy, and surgical approach, the adjusted RD was consistent with the unadjusted analysis, reinforcing the primary non-inferiority finding.

Although three patients in the TEA group discontinued protocol treatment on POD 2–3, all had received epidural analgesia through the 24-h assessment; therefore, a separate per-protocol analysis for the primary endpoint was not performed.

### Postoperative pain-related outcomes

**Fig. 3:**
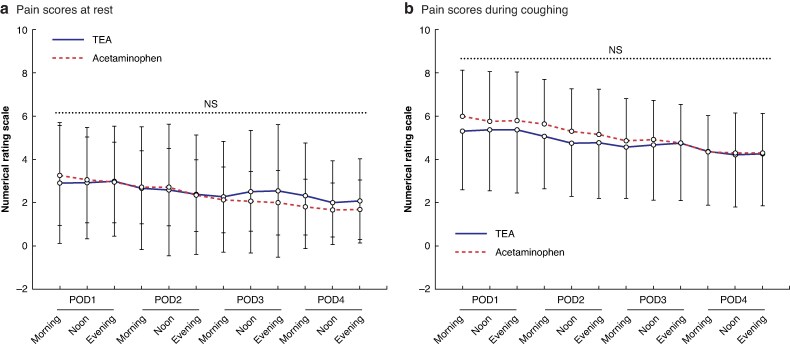
Postoperative pain intensity over time Mean numerical rating scale pain scores **a** at rest and **b** during coughing in the TEA (*n* = 68) and acetaminophen (*n* = 67) groups from POD 1 to POD 4 . Error bars indicate the standard deviation. There were no statistically significant between-group differences at any time point. TEA, thoracic epidural analgesia; POD, postoperative day; NS, not significant.

Pain trajectories at rest were similar between groups, although cough-related scores were slightly lower with TEA through POD 3 (mean difference < 1 point), converging by POD 4 (*Fig. 3*).

As summarized in *Table 2*, secondary pain outcomes, including cumulative pain burden (AUC_72_), the use of rescue doses during the first 3 days after surgery, and patient satisfaction, did not differ significantly between the two groups.

**Table 2:** Secondary pain-related outcomes (modified intention-to-treat population)

	TEA group (*n* = 68)	Acetaminophen group (*n* = 67)	Effect estimate†	P*
AUC_72_ at rest, median (i.q.r.)	154 (72–246)	140 (98–204)	4.0 (−32.0, 44.0)	0.802
AUC_72_ during coughing, median (i.q.r.)	302 (198–430)	348 (250–444)	28.0 (−20.0, 76.0)	0.268
No. of rescue doses through POD 3, median (i.q.r.)	1 (0–4)	2 (0–3)	0.0 (0.0, 1.0)	0.742
Additional opioid rescue required‡	0 (0%)	0 (0%)		
Patient satisfaction§ (% of days with score ≤ 3), median (i.q.r.)	100.0 (71.4–100.0)	85.7 (57.1–100.0)	−0.0 (−14.3, 0.0)	0.128

Values are *n* (%) unless otherwise stated. †Between-group effect estimates are presented as Hodges–Lehmann median differences with 95% confidence intervals in parentheses. ‡No patient in either group required additional opioid rescue other than that contained in the TEA regimen, which included an opioid. §Patient satisfaction was assessed once daily during POD 1–7 using a seven-point Likert scale (1 = completely satisfied; 7 = completely dissatisfied), and the proportion of days with a score ≤ 3 was calculated for each patient. Patient satisfaction was analysed in patients with complete satisfaction assessments (TEA group, *n* = 58; acetaminophen group, *n* = 59). TEA, thoracic epidural analgesia; AUC_72_, area under the curve over the first 72 hours; i.q.r., interquartile range; POD, postoperative day. *Mann–Whitney *U* test.

AUC_72_ was comparable between the two groups, both at rest and during coughing, indicating no meaningful difference in overall postoperative analgesic experience. Notably, no patient in either group required additional opioid rescue analgesia.

To complement the continuous pain trajectories, exploratory post hoc GLMMs evaluated dichotomous pain outcomes (NRS ≥ 4) at 48 and 72 h at rest, and at 24, 48, and 72 h during coughing. The adjusted RD patterns showed a small trend favouring acetaminophen at rest and TEA during coughing; however, the confidence intervals at all time points were wide and crossed zero, indicating no statistically significant difference between groups (*Fig. S1*).

Consistent results were observed in the per-protocol analysis (*Table S2*).

### Perioperative and recovery outcomes

Perioperative and recovery outcomes are summarized in *Table 3*. The time from operating room entry to incision was significantly longer in the TEA group (mean(standard deviation) 55(16) *versus* 46(17) min; *P* = 0.003), reflecting the additional time required for epidural placement. Gastrointestinal recovery (time to first flatus and defaecation) and early mobilization were comparable between the two groups. Urinary catheter removal occurred later in the TEA than acetaminophen group (median POD 3 (i.q.r. POD 3–4) *versus* POD 2 (i.q.r. POD 2–3); *P* < 0.001). The length of hospital stay was slightly shorter in the acetaminophen group, although the difference was not statistically significant.

**Table 3:** Perioperative, recovery, and safety outcomes (modified intention-to-treat population)

	TEA group (*n* = 68)	Acetaminophen group (*n* = 67)	Difference†	P*
**Perioperative parameters**				
Operating room stay (min), mean(s.d.)	333(74)	338(84)	4 (−23, 31)	0.752
Operative time (min), mean(s.d.)	249(67)	262(74)	13 (−11, 37)	0.282
Operating room entry to incision (min), mean(s.d.)	55(16)	46(17)	−9 (−14, −3)	0.003
**Recovery outcomes**				
Standing on POD 1	58 (85%)	59 (88%)	3% (−9, 14)	0.637‡
Walking on POD 1	51 (75%)	51 (76%)	1% (−13, 16)	0.880‡
Urinary catheter removal day (POD), median (i.q.r.)	3.0 (3.0–4.0)	2.0 (2.0–3.0)	−1.0 (−1.0, −1.0)	< 0.001
Time to first flatus (POD), median (i.q.r.)	2.0 (2.0–3.0)	2.0 (2.0–3.0)	0.0 (0.0, 0.0)	0.813
Time to first defecation (POD), median (i.q.r.)	4.0 (3.0–5.0)	4.0 (3.0–5.0)	0.0 (0.0, 0.0)	0.747
Postoperative hospital stay (days), median (i.q.r.)	11.0 (8.5–14.0)	10.0 (8.0–14.0)	0.0 (−1.0, 1.0)	0.489
Readmission within 30 days	1 (1.0%)	2 (3.0%)	1.5% (−3.5, 6.5)	0.551‡
**Surgical complications (Clavien–Dindo classification)**				
Grade II	10 (14.7%)	7 (10.4%)	−4.3% (−15.4, 6.9)	0.456‡
Grade III	2 (2.9%)	2 (3.0%)	0.0% (−5.7, 5.8)	> 0.999‡
**Analgesia-related adverse events**				
Postoperative nausea and vomiting	8 (11.8%)	7 (10.4%)	−1.3% (−11.9, 9.3)	> 0.999‡
Transient lower extremity paralysis	1 (1.5%)	0 (0.0%)	−1.5% (−4.3, 1.4)	> 0.999‡
**Laboratory toxicities (CTCAE)**				
AST/ALT elevation: grade 2	5 (7.4%)	7 (10.4%)	3.1% (−6.5, 12.7)	0.561‡
AST/ALT elevation: grade 3	6 (8.8%)	4 (6.0%)	−2.9% (−11.7, 6.0)	0.744‡

Values are or *n* (%) unless otherwise stated. †Values in parentheses are 95% confidence intervals. Between-group differences and corresponding 95% confidence intervals are reported to facilitate descriptive comparison. TEA, thoracic epidural analgesia; min, minutes; s.d., standard deviation; POD, postoperative day; i.q.r., interquartile range; CTCAE, Common Terminology Criteria for Adverse Events version 4.0; AST, aspartate transaminase; ALT, alanine transaminase. *Student’s *t* test or the Mann–Whitney *U* test, except ‡χ^2^ test or Fisher's exact test.

### Safety outcomes

As shown in *Table 3*, surgical complication rates were low and comparable between the two groups, with no grade IV or higher events observed in either group. Regarding analgesia-related adverse events, no serious catheter-related complications, such as epidural haematoma or abscess, were observed. One patient in the TEA group developed transient lower extremity paralysis, which resolved promptly after discontinuation of epidural analgesia. The incidence of PONV was comparable between the two groups. For laboratory toxicities, CTCAE grade 2–3 elevations in aspartate transaminase or alanine transaminase occurred at similar frequencies in both groups, indicating no evidence of increased hepatotoxicity associated with acetaminophen.

## Discussion

This multicentre pragmatic non-inferiority randomized trial evaluated analgesic strategies under routine perioperative conditions across several Japanese centres. At 24 h, the incidence of inadequate pain at rest (NRS score ≥ 4) was slightly lower in the acetaminophen group, meeting the prespecified criterion for non-inferiority to TEA. Adjusted analyses supported this finding of non-inferiority. Secondary outcomes, including cumulative pain burden (AUC_72_), the use of rescue analgesia, and patient satisfaction, were also comparable between the two groups.

A previous multicentre trial demonstrated that intravenous acetaminophen combined with TEA improved analgesia overall, with greater benefit observed in open gastrectomy^3^. The present study builds on those findings by showing that adequate analgesia can be achieved without TEA in minimally invasive gastrectomy, suggesting that routine TEA may be unnecessary in this setting.

In this study, TEA provided slightly better analgesia during coughing in the early postoperative period, suggesting that it may still offer some advantage for dynamic pain control. However, this difference was not statistically significant and consistently remained within 1 point on the NRS, well below the 2-point threshold generally considered clinically meaningful on the NRS^21^. Exploratory post hoc GLMM analyses also showed no significant differences in the proportion of patients with an NRS score ≥ 4 at rest or during coughing throughout POD 3. The time from operating room entry to incision was longer in the TEA group, reflecting the additional procedural time required for epidural catheter placement, which may have implications for operating room efficiency. Importantly, postoperative recovery profiles were comparable between the two groups, although urinary catheter removal occurred earlier in the acetaminophen group. Beyond these clinical outcomes, the use of TEA presents several challenges, such as the technical skill required for catheter insertion and its well recognized adverse effects. Furthermore, as the cancer population continues to age and the use of anticoagulants becomes more prevalent among elderly patients, the perioperative management of TEA has become substantially more complex^22,23^. Together, the findings of minimal differences in dynamic pain, comparable recovery outcomes, and the procedural and logistical burdens associated with TEA support the use of a simple acetaminophen-based regimen as a practical and reliable option for minimally invasive gastrectomy.

A recent single-centre RCT by Kikuchi *et al*.^24^ did not demonstrate non-inferiority of opioid-based patient-controlled intravenous analgesia to TEA after laparoscopic gastrectomy. Differences between the present study and that of Kikuchi *et al*.^24^ in analgesic regimen and endpoint selection, as well as the pragmatic multicentre design of the present study, may help explain the differences in results. In addition, all patients in the present study received standardized wound infiltration at skin closure, ensuring consistent short-term local analgesia across groups and representing an important methodological distinction from previous studies.

The choice of primary endpoint also warrants comment. In the present study, an NRS score ≥ 4 at rest 24 h after surgery was selected as the threshold for inadequate pain because, according to the National Comprehensive Cancer Network Guidelines^25^, this level corresponds to moderate or greater pain requiring clinical intervention. The 24-h timepoint captures the period when pain is typically most intense and when effective control is crucial to support early mobilization within ERAS programs. Thus, this standardized, guideline-based threshold provides a clinically meaningful and interpretable measure of analgesic efficacy.

Safety outcomes were likewise comparable between the TEA and acetaminophen groups. The rates and severity of postoperative complications were similar between the two groups, and no evidence of acetaminophen-related hepatotoxicity was observed. One patient in the TEA group developed transient motor weakness that resolved promptly after catheter removal. Together, these findings indicate that scheduled intravenous acetaminophen is a safe alternative to TEA for minimally invasive gastrectomy.

From a broader perspective, the results of the present study align with the global shift towards simplified opioid-sparing perioperative care. Previous studies in less invasive surgeries for benign disease have shown that opioid-free or opioid-sparing anaesthesia can reduce PONV while maintaining adequate pain control^26–28^. Demonstrating this approach in major oncological surgery is clinically meaningful because even brief perioperative opioid exposure may increase the risk of prolonged use. In the present cohort, no patient required postoperative rescue opioids, highlighting that effective analgesia can be achieved without routine reliance on TEA or opioid-based regimens when multimodal strategies are optimized.

Several study limitations should be acknowledged. First, enrolment across seven institutions over a prolonged period may have introduced heterogeneity in surgical and anaesthetic management, which is an inherent limitation of pragmatic multicentre trials. Conversely, this design reflects real-world clinical settings and enhances external validity. Baseline characteristics were well balanced, and the primary non-inferiority finding remained consistent after adjustment for institution and surgical factors. Second, surgical approach (laparoscopic or robot-assisted) was not specified as a minimization factor in the protocol. Because surgical approach may potentially influence postoperative pain, an additional adjusted analysis was performed including this variable. The distribution of surgical approach was well balanced between groups, and the adjusted results were consistent with the primary analysis. Third, blinding of patients and clinicians was not feasible, but the primary endpoint was a standardized pain measure, and statisticians were blinded to treatment allocation. Fourth, this study evaluated only short-term postoperative outcomes, precluding assessment of longer-term measures such as chronic pain, quality of life, and cost-effectiveness.

In conclusion, this is the first pragmatic multicentre non-inferiority RCT in minimally invasive gastrectomy to demonstrate that a simple and reproducible non-epidural regimen, namely scheduled intravenous acetaminophen combined with local wound infiltration, met the prespecified criterion for non-inferiority to TEA for postoperative pain control. Together, the findings of the present study support the use of an acetaminophen-based multimodal regimen as a pragmatic, non-epidural alternative to TEA within ERAS protocols, and may help inform analgesic pathways for gastric cancer surgery in the minimally invasive era.

## Supplementary Material

zrag031_Supplementary_Data

## Data Availability

Deidentified participant data are available from the corresponding author upon reasonable request. Due to institutional and ethical restrictions, individual-level data cannot be made publicly available.
