# Supplementary material for: Scheduled intravenous acetaminophen versus thoracic epidural analgesia for postoperative pain control after minimally invasive gastrectomy: multicentre randomized non-inferiority trial
Source: BJS Open. 2026 Apr 27;10(2):zrag031. doi: 10.1093/bjsopen/zrag031 (PMC13111924; doi:10.1093/bjsopen/zrag031)
Supplement: zrag031_Supplementary_Data [file zrag031_supplementary_data.zip › Study Protocol.docx]

**Scheduled intravenous acetaminophen versus thoracic epidural analgesia after minimally invasive gastrectomy: A multicenter, randomized, non-inferiority trial**

**Study Protocol****Version 6 (Final, February 2, 2023)**

**Principal Investigator:**

Jun Kinoshita, MD, PhD

Department of Gastrointestinal Surgery, Kanazawa University Hospital, Kanazawa, Japan

Adress: 13-1 Takaramachi, Kanazawa, Japan
Tel: +81-76-265-2362

E-mail: junkino@staff.kanazawa-u.ac.jp

**Study Group Representative:**

Noriyuki Inaki, MD, PhD

Chair, Gastrointestinal Cancer Conference (GICC) Study Group

Department of Gastrointestinal Surgery, Kanazawa University Hospital, Kanazawa, Japan

**Data Management and Coordination:**

Sachio Fushida, MD, PhD

Digestive Disease Support Organization (DDSO), Kanazawa, Japan

Department of Gastrointestinal Surgery, Kanazawa University Hospital, Kanazawa, Japan

**Protocol Version:**

Version 1 First edition (February 26, 2020)

Version 2 (September 25, 2021)

Version 3 (December 28, 2021)

Version 4 (March 8, 2022)

Version 5 (January 11, 2023)

Version 6 (February 2, 2023).

Table of contents

[**0. Study Summary** 5](#_Toc214471904)

[0.1 Objectives 5](#_Toc214471905)

[0.2 Study Design 5](#_Toc214471906)

[0.3 Eligibility Criteria 5](#_Toc214471907)

[0.4 Exclusion Criteria 6](#_Toc214471908)

[0.5 Main Outcome 6](#_Toc214471909)

[0.6 Target Number of Patients 6](#_Toc214471910)

[0.7 Research Period 7](#_Toc214471911)

[**1. Objective** 8](#_Toc214471912)

[**2. Background and Scientific Rationale** 8](#_Toc214471913)

[2.1 Background 8](#_Toc214471914)

[2.2 Scientific Rationale 10](#_Toc214471915)

[**3. Investigational Drug Information** 10](#_Toc214471916)

[3.1 Study Drug 10](#_Toc214471917)

[**4. Participants** 11](#_Toc214471918)

[4.1 Eligibility Criteria 11](#_Toc214471919)

[4.2 Exclusion Criteria 12](#_Toc214471920)

[**5. Patient Enrollment and Randomization** 12](#_Toc214471921)

[5.1 Patient Registration Procedure 12](#_Toc214471922)

[5.2 Randomization Method and Stratification Factors 13](#_Toc214471923)

[**6. Study plan** 13](#_Toc214471924)

[**6.1 Protocol treatment** 14](#_Toc214471925)

[6.2 Criteria for Dose or Schedule Modification 15](#_Toc214471926)

[6.3 Discontinuation of Study Treatment 16](#_Toc214471927)

[6.4 Concomitant and Supportive Therapy 16](#_Toc214471928)

[6.5 Post-Study Treatment 17](#_Toc214471929)

[**7. Schedule of Observations, Assessments, and Reporting** 17](#_Toc214471930)

[7.1 Preoperative Assessments 17](#_Toc214471931)

[7.2 Intraoperative Assessments 17](#_Toc214471932)

[7.3 Postoperative Assessments 17](#_Toc214471933)

[7.4 Adverse Event Monitoring 18](#_Toc214471934)

[**8. Target Sample Size and Study Period** 18](#_Toc214471935)

[8.1 Target Sample Size 18](#_Toc214471936)

[8.2 Study Period 18](#_Toc214471937)

[**9. Evaluation and Reporting of Adverse Events** 18](#_Toc214471938)

[9.1 Definition of Adverse Events 18](#_Toc214471939)

[9.2 Evaluation and Reporting of Adverse Events 19](#_Toc214471940)

[9.3 Expected Adverse Events 20](#_Toc214471941)

[9.4 Reporting and Management of Serious Adverse Events 21](#_Toc214471942)

[9.5 Efficacy and Safety Evaluation Committee 22](#_Toc214471943)

[**10. Benefits and Disadvantages of Participation** 23](#_Toc214471944)

[10.1 Potential Risks 23](#_Toc214471945)

[10.2 Potential Benefits 23](#_Toc214471946)

[10.3 Comprehensive Risk Assessment and Measures to Minimize Burden and Risk 23](#_Toc214471947)

[**11. Reporting to the Institutional Director** 24](#_Toc214471948)

[**12. Definition of Endpoints** 25](#_Toc214471949)

[12.1 Primary Endpoint 25](#_Toc214471950)

[12.2 Secondary Endpoints 25](#_Toc214471951)

[**13. Statistical Analysis** 25](#_Toc214471952)

[13.1 Sample Size Determination 25](#_Toc214471953)

[13.2 Analysis Populations 26](#_Toc214471954)

[13.3 Interim Analysis 26](#_Toc214471955)

[13.4 Analysis Variables and Statistical Methods 26](#_Toc214471956)

[**14. CRFs: Completion and Submission** 27](#_Toc214471957)

[**15. Monitoring** 28](#_Toc214471958)

[**16. Ethical Considerations** 28](#_Toc214471959)

[16.1 Regulatory and Ethical Compliance 28](#_Toc214471960)

[16.2 Preparation and Revision of the Informed Consent Documents 29](#_Toc214471961)

[16.3 Informed Consent 29](#_Toc214471962)

[**17. Protection of Personal Information** 30](#_Toc214471963)

[**18. Funding, Conflict of Interest, and Compensation** 31](#_Toc214471964)

[18.1 Funding Source and Financial Relationships 31](#_Toc214471965)

[18.2 Study-Related Costs 31](#_Toc214471966)

[18.3 Compensation for Health-Related Injury 31](#_Toc214471967)

[**19. Protocol Deviations, Amendments, and Revisions** 31](#_Toc214471968)

[19.1 Protocol Deviations or Modifications 31](#_Toc214471969)

[19.2 Protocol Revisions 32](#_Toc214471970)

[**20. Study Completion and Early Termination** 32](#_Toc214471971)

[20.1 Study Completion 32](#_Toc214471972)

[20.2 Early Termination of the Study 32](#_Toc214471973)

[**21. Management and Retention of Study Documents** 33](#_Toc214471974)

[**22. Ownership and Publication of Study Results** 33](#_Toc214471975)

[**23. Protocol Amendments Summary** 33](#_Toc214471976)

[**24. participating trial centers** 34](#_Toc214471977)

[**25. References** 35](#_Toc214471978)

**0. Study Summary**

**0.1 Objectives**

To determine whether a scheduled intravenous acetaminophen–based multimodal analgesic regimen is non-inferior to TEA for postoperative pain control after minimally invasive gastrectomy for gastric cancer.

**0.2 Study Design**


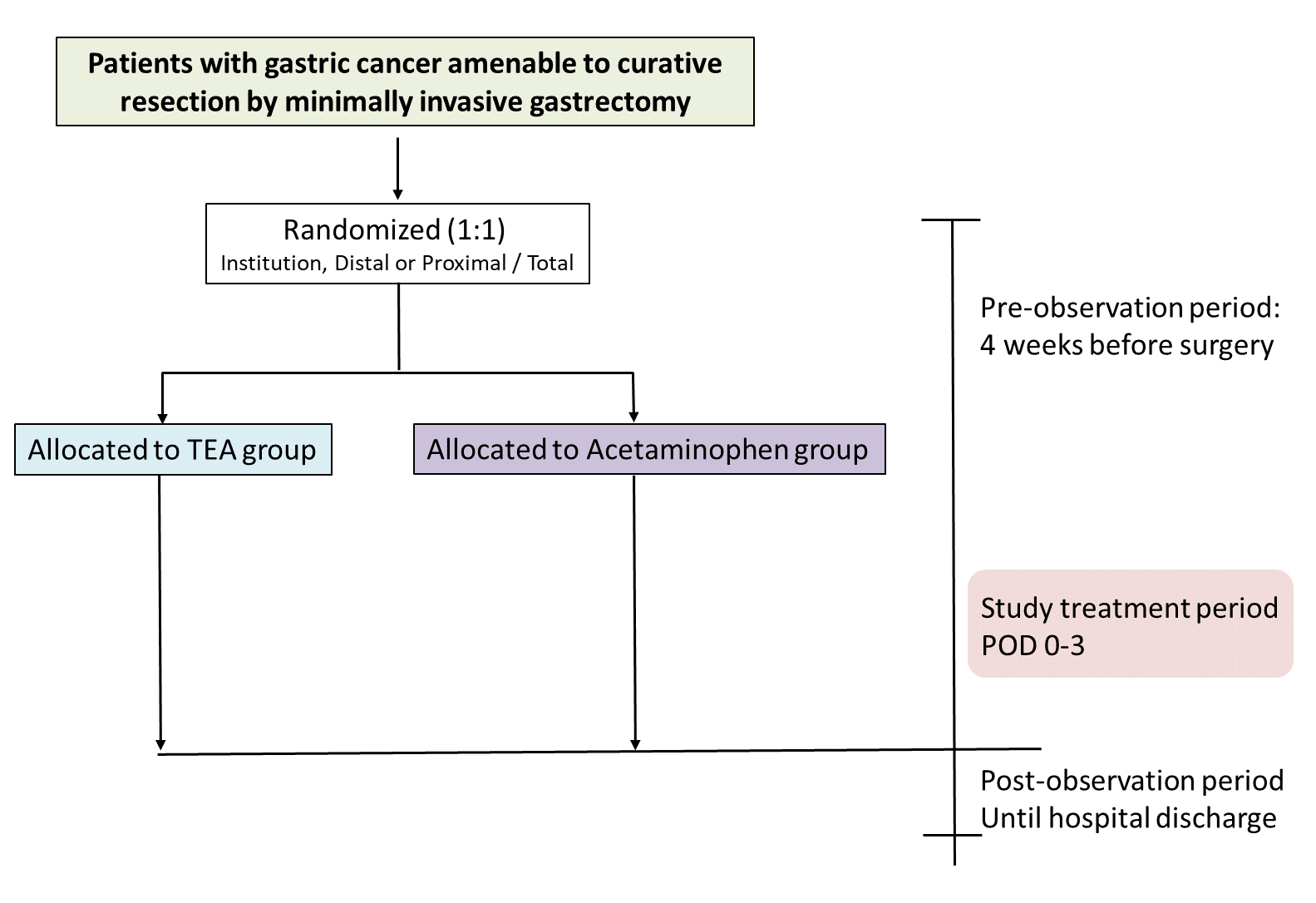
This study is a pragmatic, multicenter, randomized, open-label, parallel-group, non-inferiority trial.

### **0.3 Eligibility Criteria**

1. Age ≥ 20 years at the time of consent.
2. Histologically confirmed gastric adenocarcinoma.
3. Clinical stage I–III disease (as defined by the Japanese Classification of Gastric Carcinoma, 3rd English edition [10]).
4. Scheduled to undergo curative minimally invasive gastrectomy (laparoscopic or robot-assisted) with D1+ or D2 lymph node dissection.
5. ECOG performance status 0–1.
6. Adequate organ function confirmed within 4 weeks before surgery, including hematologic, hepatic, and renal parameters within institutional reference ranges:
    • White blood cell count ≥ 3,000 / μL
    • Platelet count ≥ 100,000 / μL
    • AST ≤ 100 U/L
    • ALT ≤ 100 U/L
    • Total bilirubin ≤ 2.0 mg/dL
    • Serum creatinine ≤ 1.5 mg/dL
7. Written informed consent is obtained prior to participation.

### **0.4 Exclusion Criteria**

1. Active double cancer requiring treatment at enrollment.
2. Contraindication to thoracic epidural anesthesia (e.g., coagulopathy, bleeding tendency, increased intracranial pressure).
3. Pulmonary disease of Hugh–Jones grade II or higher.
4. Unstable angina (newly developed or worsened within 3 weeks) or recent myocardial infarction (< 6 months).
5. Poorly controlled hypertension or diabetes mellitus.
6. History of hypersensitivity to acetaminophen or ropivacaine hydrochloride hydrate (Anapeine®) or other amide-type local anesthetics.
7. Aspirin-induced asthma or bronchospasm triggered by NSAIDs.
8. Chronic systemic corticosteroid therapy (oral or intravenous).
9. Pregnancy or breastfeeding, or possible pregnancy.
10. Any other condition that, in the judgment of the investigator, makes the patient unsuitable for participation.

**0.5 Main Outcome**

The proportion of patients with a Numerical Rating Scale (NRS) score ≥ 4 at rest 24 hours after surgery.

**0.6 Target Number of Patients**

A total of 140 patients will be enrolled (70 per group) across institutions belonging to the GICC study group.

**0.7 Research Period**

From February 27, 2020 to January 31, 2025.

**ABBREVIATIONS**

| **TEA** | Thoracic epidural analgesia |
| --- | --- |
| **ERAS** | Enhanced Recovery After Surgery |
| **NSAIDS** | Nonsteroidal anti-inflammatory drugs |
| **PONV** | Postoperative nausea and vomiting |
| **CRF** | Case report form |
| **NRS** | Numerical rating scale |
| **POD** | Postoperative day |
| **IRB** | Institutional review board |
| **UMIN** | University Hospital Medical Information Network |
| **RCT** | Randomized controlled trial |

**1. Objective**

To evaluate, in a multicenter randomized controlled non-inferiority trial, whether scheduled intravenous acetaminophen provides postoperative analgesia that is non-inferior to the standard treatment with thoracic epidural analgesia (TEA) in patients undergoing gastrectomy.

**2. Background and Scientific Rationale**

2.1 Background

**2.1.1 Target disease and surgical setting**

Patients with gastric cancer amenable to curative resection by minimally invasive gastrectomy.

**2.1.2 Current standard treatment**

In Japan, perioperative management for gastric cancer surgery is largely standardized through institutional clinical pathways; however, no universally established standard exists for postoperative pain management. Optimal pain control is crucial for preventing complications involving the cardiovascular, respiratory, and neuropsychiatric systems. In particular, wound pain after upper abdominal surgery such as gastrectomy has a marked impact on the cardiopulmonary system. TEA reduces the intraoperative anesthetic requirement, promotes bowel peristalsis, and provides effective postoperative analgesia [1]. Owing to these advantages, TEA is routinely performed for open gastrointestinal surgery in most institutions in Japan. In a previous questionnaire survey conducted across 14 participating institutions in this study, TEA was also used for laparoscopic or robotic gastrectomy, despite its relatively mild wound pain compared with open procedures.

**2.1.3 Limitations of current practice and emerging alternatives**

Although TEA offers multiple benefits, its postoperative use has recently declined because of (1) the increasing number of patients requiring postoperative anticoagulant therapy, (2) the widespread adoption of minimally invasive surgery with smaller incisions, and (3) advances in alternative analgesic methods such as intravenous patient-controlled analgesia and peripheral nerve blocks.

TEA carries a risk of epidural hematoma during catheter placement or removal, particularly in patients receiving anticoagulation therapy. If hematoma occurs, surgical decompression within 8 hours is essential to prevent irreversible neurological injury; although the incidence is rare (~0.01%), it is a potentially catastrophic complication. TEA also increases the risk of urinary retention, delaying catheter removal and ambulation, and can cause adverse effects such as nausea or pruritus associated with epidurally administered opioids [2,3]. Given Japan’s rapidly aging population and the expected increase in elderly gastric cancer patients on anticoagulant therapy, developing a safe and effective alternative to TEA for postoperative analgesia is a pressing need.

**2.1.4 Enhanced Recovery After Surgery (ERAS) framework**

The ERAS program is a multidisciplinary perioperative care pathway aimed at enhancing postoperative recovery, reducing complications, shortening hospital stay, and lowering healthcare costs [4]. In the consensus statement on anesthetic management for gastrointestinal surgery within the ERAS protocol, multimodal analgesia combining scheduled administration of nonsteroidal anti-inflammatory drugs (NSAIDs), COX-2 inhibitors, and acetaminophen is recommended to improve analgesic efficacy and minimize opioid use. Among these, acetaminophen is expected to be particularly effective as it is associated with a low incidence of severe adverse events such as gastrointestinal ulceration and renal dysfunction, and has been reported to reduce postoperative nausea and vomiting (PONV) related to opioid use [5].

While current ERAS Society guidelines for gastrectomy recommend TEA for open procedures, supporting evidence for minimally invasive gastrectomy remains limited, and no procedure-specific analgesic protocol has yet been established [6].

Previously, our study group conducted a multicenter randomized controlled trial comparing TEA alone (the current standard) with TEA plus scheduled intravenous acetaminophen after gastrectomy [7]. The trial demonstrated that the addition of acetaminophen significantly improved postoperative pain control. However, subgroup analyses revealed that this additive effect was evident only in open gastrectomy, whereas no benefit was observed in laparoscopic procedures. These findings suggest that in minimally invasive surgery, where wound pain is mild, scheduled acetaminophen may not confer additional analgesic benefit when combined with TEA.

## 2.2 Scientific Rationale

**2.2.1 Previous evidence**

Two clinical studies have evaluated the impact of TEA on postoperative analgesia after laparoscopic gastrectomy.　One randomized controlled trial　(RCT) comparing TEA with intravenous opioid analgesia after laparoscopic gastrectomy reported faster bowel recovery and slightly lower early pain scores with TEA, it showed no improvement in overall recovery [8].

Another single-center prospective study compared patients receiving scheduled oral analgesics (tramadol, acetaminophen, NSAIDs, or prochlorperazine maleate) without TEA (n = 34) with a historical TEA control group (n = 34). The frequency of additional rescue analgesics was significantly lower in the non-TEA group (1 vs 3 times, p = 0.006) [9]. Both studies were single-institution trials; to date, no RCT has evaluated the impact of omitting TEA on postoperative analgesia after minimally invasive gastrectomy.

**2.2.2 Rationale for the present study**

Therefore, this study was designed to develop a TEA-free postoperative analgesic strategy for patients undergoing minimally invasive gastrectomy, in whom wound pain is generally mild. We aim to evaluate, through a multicenter randomized non-inferiority trial, whether scheduled intravenous acetaminophen provides postoperative analgesia that is non-inferior to standard TEA. To ensure adequate immediate postoperative pain relief, local wound infiltration anesthesia will be performed in both groups at the end of surgery.

# **3. Investigational Drug Information**

## 3.1 Study Drug

**3.1.1 Investigational drug**

Acetaminophen intravenous infusion (Acelio® Injection 1000 mg; Acetaminophen Injection)

**3.1.2 Manufacturer / distributor**

Terumo Corporation, Tokyo, Japan

**3.1.3 Mechanism of action**

Refer to the package insert for detailed pharmacologic information.

**3.1.4 Indications**

Relief of pain and reduction of fever in cases where oral or suppository administration is not feasible.

**3.1.5 Dosage and administration (according to the package insert)**

For adults, the usual dose of acetaminophen is 300–1000 mg administered intravenously over 15 minutes at intervals of at least 4 to 6 hours. The dosage may be adjusted according to age and symptoms, but the maximum total daily dose should not exceed 4000 mg.　For adults weighing less than 50 kg, the recommended dose is up to 15 mg/kg per dose, administered intravenously over 15 minutes at intervals of at least 4 to 6 hours, with a maximum total daily dose of 60 mg/kg.

**4. Participants**

**4.1 Eligibility Criteria**

Participants are eligible for enrollment if they meet all of the following conditions:

1. Age ≥ 20 years at the time of consent.
2. Histologically confirmed gastric adenocarcinoma.
3. Clinical stage I–III disease (as defined by the Japanese Classification of Gastric Carcinoma, 3rd English edition [10]).
4. Scheduled to undergo curative minimally invasive gastrectomy (laparoscopic or robot-assisted) with D1+ or D2 lymph node dissection.
5. ECOG performance status 0–1.
6. Adequate organ function confirmed within 4 weeks before surgery, including hematologic, hepatic, and renal parameters within institutional reference ranges:
    • White blood cell count ≥ 3,000 / μL
    • Platelet count ≥ 100,000 / μL
    • AST ≤ 100 U/L
    • ALT ≤ 100 U/L
    • Total bilirubin ≤ 2.0 mg/dL
    • Serum creatinine ≤ 1.5 mg/dL
7. Written informed consent is obtained prior to participation.

**4.2 Exclusion Criteria**

Patients are excluded if any of the following apply:

1. Active double cancer requiring treatment at enrollment.
2. Contraindication to thoracic epidural anesthesia (e.g., coagulopathy, bleeding tendency, increased intracranial pressure).
3. Pulmonary disease of Hugh–Jones grade II or higher.
4. Unstable angina (newly developed or worsened within 3 weeks) or recent myocardial infarction (< 6 months).
5. Poorly controlled hypertension or diabetes mellitus.
6. History of hypersensitivity to acetaminophen or ropivacaine hydrochloride hydrate (Anapeine®) or other amide-type local anesthetics.
7. Aspirin-induced asthma or bronchospasm triggered by NSAIDs.
8. Chronic systemic corticosteroid therapy (oral or intravenous).
9. Pregnancy or breastfeeding, or possible pregnancy.
10. Any other condition that, in the judgment of the investigator, makes the patient unsuitable for participation.

Rationale: Criterion 1 ensures unbiased efficacy evaluation and patient safety; Criteria 2–10 are established primarily for safety considerations.

# **5. Patient Enrollment and Randomization**

## 5.1 Patient Registration Procedure

Patient registration in this study will be conducted centrally by the data center.

(1) The principal investigator or a sub-investigator will obtain written informed consent from each eligible patient.

(2) After obtaining consent, the investigator will record the required information (date of consent, subject identification code, patient name, medical record number, etc.) on the subject identification code list maintained by the personal information manager. The personal information manager will securely store this list in a locked location under their control.

(3) After confirming that all inclusion criteria are met and that none of the exclusion criteria apply, the investigator will complete the case registration form including the subject identification code and submit it to the data center.

(4) The data center will verify eligibility. If the eligibility criteria are satisfied, the data center will issue a registration confirmation sheet that specifies the registration number and the allocated treatment group, and will notify the principal investigator or the responsible investigator.

(5) Upon receipt of the registration confirmation sheet, the investigator will confirm its contents and initiate the study intervention.

(6) In the event of withdrawal of consent, discontinuation, or dropout, the investigator must promptly report the details to the principal investigator.

## 5.2 Randomization Method and Stratification Factors

At the time of registration, each subject will be randomly assigned to a treatment group (1:1) by GICC data center. Randomization will be performed using the minimization method, with the following stratification factors to ensure balance between groups:

(1) participating institution,

(2) type of gastrectomy (distal vs proximal or total gastrectomy)

The detailed randomization procedure and algorithm will not be disclosed to investigators at participating institutions.

**6. Study plan**

**6.1 Protocol treatment**

**6.1.1 Study outline**

**
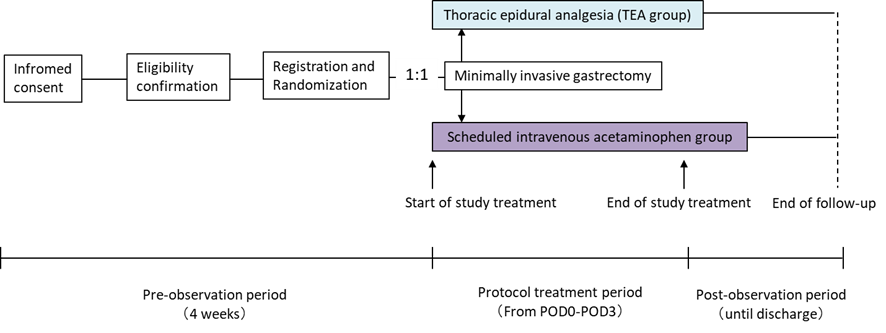
**

At the time of consent, baseline examinations will be performed, followed by preoperative assessments conducted immediately before surgery. The pre-observation period from consent to surgery will be limited to 4 weeks. If eligibility criteria are not met at the final preoperative assessment, the patient will be excluded before surgery.

Patients scheduled to undergo minimally invasive gastrectomy for curative resection of gastric cancer who meet all inclusion criteria and provide written informed consent will be registered and randomized by the data center.

- Group A (TEA group): Patients will receive thoracic epidural analgesia intraoperatively, which will also be used for postoperative pain management.
- Group B (Acetaminophen group): Patients will not receive epidural anesthesia but will instead receive scheduled intravenous acetaminophen for three postoperative days.

**6.1.2 Details of intervention**

Group A –TEA Group

An epidural catheter is placed at the thoracic level (T8–T10) before induction of anesthesia.The choice and dosage of anesthetic agents are determined according to each institution’s policy; however, each institution applies a consistent regimen across all enrolled patients. At Kanazawa University Hospital, the standard epidural solution consists of: 0.2% ropivacaine (Anapeine®) 280–290 mL + 0.005% fentanyl 10–20 mL (total 300 mL).
Continuous infusion with optional patient-controlled epidural analgesia is permitted to maintain an numerical rating scale (NRS) < 4 at rest.
The epidural catheter is removed after the infusion is completed, generally by postoperative day (POD) 3.
If pain is inadequately controlled or at the patient’s request, additional epidural boluses are permitted and recorded in the case report form (CRF).

Group B – Scheduled Intravenous Acetaminophen Group

Following surgery, Acelio® IV infusion (Terumo Corporation, Tokyo, Japan) is administered immediately after completion of surgery and repeated every 6 hours until POD 3—
1000 mg per dose for patients weighing ≥ 50 kg, or 15 mg/kg per dose for those weighing < 50 kg—
not exceeding the approved maximum daily dosage in Japan.

Common Perioperative Management

All patients receive intraoperative local wound infiltration with 10 mL of 0.75% ropivacaine diluted with 10 mL of saline, which is injected into the subfascial (ie, pre-peritoneal) space under direct vision at skin closure.
Routine perioperative care, including fluid management, early mobilization, and diet advancement, follows institutional ERAS-based protocols.
Rescue analgesics for breakthrough pain (NRS ≥ 4) are allowed at the discretion of the attending physician and recorded in the CRF. The type and frequency of rescue medication are documented.

## 6.2 Criteria for Dose or Schedule Modification

If adverse events such as hypotension or pruritus occur due to TEA, the attending physician may temporarily interrupt epidural infusion. Re-administration may be resumed once the symptoms have resolved. Any temporary interruption or permanent discontinuation must be documented in the CRF.

## 6.3 Discontinuation of Study Treatment

The principal investigator or sub-investigator shall discontinue study treatment when continuation is deemed inappropriate for any of the following reasons.
The date, reason, and clinical course will be recorded in the medical chart and CRF, and necessary assessments will be performed at the time of discontinuation to evaluate efficacy and safety.
If treatment is discontinued due to an adverse event, the subject will be followed until recovery whenever possible.

Reasons for discontinuation:

1. The participant withdraws consent or requests to discontinue participation.
2. The participant is found post-registration not to meet the eligibility criteria.
3. The participant is diagnosed as clinical Stage IV based on intraoperative findings.
4. Conversion to open surgery is required due to intraoperative findings or complications.
5. Continuation of the protocol treatment becomes difficult because of intraoperative complications.
6. The study cannot continue due to worsening postoperative complications.
7. Continuation is deemed difficult because of adverse events.
8. Pregnancy is identified after enrollment.
9. The entire study is terminated.
10. The investigator determines that discontinuation is appropriate for any other reason.

## 6.4 Concomitant and Supportive Therapy

Prohibited concomitant medications:

Group B: Other drugs containing acetaminophen.

Both groups: Routine postoperative use of oral or intravenous opioids as rescue analgesia is in principle prohibited. If pain control is extremely inadequate, the attending physician may consider opioid use; in such cases, the type of opioid, dose, and frequency must be recorded in the CRF.

## 6.5 Post-Study Treatment

From POD 4 onward, postoperative pain management is performed at the discretion of each attending physician, according to institutional standards.

**7. Schedule of Observations, Assessments, and Reporting**

**7.1 Preoperative Assessments**

Before enrollment, the following baseline evaluations are performed to confirm eligibility and general condition:
• Physical examination and ECOG performance status.
• Anthropometrics (height, weight).
• Complete blood count and serum biochemistry (total protein, albumin, electrolytes, bilirubin, AST, ALT, creatinine, amylase, CRP).
• Upper abdominal and pelvic CT scan.
• Upper gastrointestinal endoscopy with biopsy.
• Chest X-ray, 12-lead ECG, and pulmonary function test (FEV1.0%, %VC).

**7.2 Intraoperative Assessments**

• Surgical procedure (distal, or proximal / total gastrectomy).
• Laparoscopic or robotic approach.
• Duration of surgery and anesthesia.
• Extent of lymph node dissection and reconstruction method.
• Intraoperative blood loss and blood transfusion status (yes/no).

**7.3 Postoperative Assessments**

• Pathological evaluation: histological stage and residual tumor status.
• Postoperative complications: evaluated until discharge; graded by Clavien–Dindo [12]and CTCAE v4.0.
• Pain-related adverse events: hypotension, bradycardia, urinary retention, nausea, vomiting, hepatic dysfunction (graded by CTCAE v4.0).
• Laboratory tests: POD 1, 3, and 7 (CBC and serum biochemistry).
• Recovery outcomes: days to first flatus/defecation, ambulation, fever (max temp POD1–7), inflammatory markers (max WBC, CRP POD1–7), hospital stay, rescue analgesics.
• Pain assessment: NRS (rest and cough) three times daily (morning, noon, evening) POD1–7.
• Patient satisfaction: assessed daily POD1–7 using a structured questionnaire.

**7.4 Adverse Event Monitoring**

All adverse events, including abnormal laboratory findings, are recorded in CRFs with details of onset, resolution, severity, treatment, and causality.
Surgical complications are classified by Clavien–Dindo; non-surgical events are graded by CTCAE v4.0.

**8. Target Sample Size and Study Period**

**8.1 Target Sample Size**

A total of 140 patients will be enrolled (70 per group) across institutions belonging to the GICC study group.

**8.2 Study Period**

• Enrollment period: February 27, 2020 – January 31, 2025
• Observation: 8 weeks after last enrollment
• Follow-up: 4 weeks after observation
• Total duration: 6 years, until April 30, 2025

# **9. Evaluation and Reporting of Adverse Events**

## 9.1 Definition of Adverse Events

An *adverse event (AE)* is defined as any unfavorable or unintended sign, symptom, or disease (including abnormal laboratory findings) occurring in a study participant, regardless of whether it has a causal relationship with the study treatment.

A *serious adverse event (SAE)* refers to any AE that results in one or more of the following outcomes:

1. death,
2. a life-threatening condition,
3. hospitalization or prolongation of an existing hospitalization,
4. persistent or significant disability or incapacity, or
5. a congenital anomaly or birth defect in offspring.

An *unexpected serious adverse event (USAE)* is defined as an SAE whose nature or severity is not consistent with the information described in the study protocol, informed consent form, or other study-related documentation.

## 9.2 Evaluation and Reporting of Adverse Events

The evaluation of adverse events will be performed according to the Clavien-Dindo classification the Common Terminology Criteria for Adverse Events (CTCAE), version 4.0. Grading will be assigned based on the definition that most closely matches the clinical presentation (Grades 1–4). When specific management actions are indicated in the grading criteria, grading will also consider the clinical necessity of such interventions.

The principal investigator or attending physician shall take appropriate medical action upon identifying an AE and report it in CRF. Regardless of its causal relationship to the study intervention, each AE will be monitored throughout the study period and followed until resolution whenever possible.

All AEs occurring within 4 weeks after completion or discontinuation of the protocol treatment will be recorded and assessed, irrespective of causality.
However, the following will be excluded from the scope of evaluation in this study:

- AEs that occur before the administration of the epidural or local infiltration anesthetic during surgery, and
- AEs that newly occur or worsen after the initiation of post-study treatment.

AEs occurring beyond 4 weeks after completion or discontinuation of the study treatment will be observed as far as possible if considered potentially related to the investigational drug.
In cases where long-term follow-up is impractical—such as symptom chronicity due to progression of the underlying disease, transfer to another hospital, or initiation of subsequent therapy—continued observation will not be required.

The principal investigator shall submit an annual report to the hospital director summarizing the progress of the study and the occurrence of AEs or other safety-related events associated with study implementation.

## 9.3 Expected Adverse Events

(1) Intravenous Acetaminophen (Acelio® Injection 1000 mg)

The following adverse reactions have been reported in association with intravenous acetaminophen:

- Hypersensitivity reactions: shock and anaphylaxis presenting with dyspnea, flushing, urticaria, or angioedema (frequency unknown).
- Severe cutaneous reactions: toxic epidermal necrolysis (TEN), Stevens–Johnson syndrome, and acute generalized exanthematous pustulosis (frequency unknown).
- Hepatic dysfunction: fulminant hepatitis, elevated liver enzymes (AST, ALT, γ-GTP), and jaundice (frequency unknown).
- Hematologic reactions: agranulocytosis (frequency unknown).
- Respiratory reactions: asthma attacks or interstitial pneumonia (frequency unknown).
- Renal disorders: interstitial nephritis and acute renal failure (frequency unknown).
- Drug-induced hypersensitivity syndrome (frequency unknown).

Close monitoring is required during administration, and the study drug should be discontinued immediately if any abnormal signs or symptoms are observed.

(2) Ropivacaine (Anapeine® Injection 7.5 mg/mL)

Adverse events associated with ropivacaine include:

- Anaphylactic or circulatory reactions: hypotension, bradycardia, arrhythmia, respiratory depression, or loss of consciousness; in rare cases, cardiac arrest may occur.
- Central nervous system toxicity: dizziness, tremor, or convulsions due to overdose or accidental intravascular injection (incidence <0.1%).
- Neurologic complications: transient paresthesia or, rarely, persistent sensory or motor impairment, pain, or bladder/rectal dysfunction due to nerve injury or ischemia (incidence <1%).

Patients should be observed carefully during and after administration, and appropriate treatment should be initiated immediately if adverse reactions are suspected.

## 9.4 Reporting and Management of Serious Adverse Events

**9.4.1 Reporting of Serious Adverse Events**

If a serious adverse event (SAE) occurs, the sub-investigator shall take all necessary medical actions and immediately report the event to the principal investigator, irrespective of its causal relationship to the study treatment.
The principal investigator will promptly report the SAE to the director of the study institution and notify the principal investigators or coordinating investigators of collaborating institutions, as well as the study drug provider. The event will also be reported to the Efficacy and Safety Evaluation Committee, if deemed necessary.

The institutional director will review the report, seek the opinion of the institutional ethics review board, and take appropriate actions.
The reporting format and procedures will comply with each institution’s *Standard Operating Procedures for Serious Adverse Events*.

Emergency contact:
Study Group Representative: Prof. Noriyuki Inaki
Department of Gastrointestinal Surgery, Kanazawa University Hospital
13-1 Takara-machi, Kanazawa, Japan
Tel: +81-76-265-2362 Fax: +81-234-4260
(After hours / holidays) Department of Gastrointestinal Surgery, Kanazawa University Hospital
Tel: +81-76-265-2362 Fax: +81-76-265-2743

**9.4.2 Reporting to Regulatory Authorities**

When an SAE occurs, the principal investigator will promptly assess its expectedness and causal relationship to the study intervention.
If the event is determined to be an unexpected serious adverse event (USAE) and a causal relationship to the study cannot be excluded, the institutional director shall, without delay, submit a report to the Minister of Health, Labour and Welfare of Japan using the designated Unexpected Serious Adverse Event Report form, in accordance with national ethical guidelines.
The status of the event and the measures taken will also be made publicly available as appropriate.

**9.4.3 Detailed and Follow-up Reporting**

The principal investigator will provide a detailed SAE report to the institutional director and notify the study drug provider.
The report format and procedures will follow each institution’s *Standard Operating Procedures for Serious Adverse Events*.

## 9.5 Efficacy and Safety Evaluation Committee

An independent Efficacy and Safety Evaluation Committee will be established by the principal investigator to evaluate study progress, safety data, and major efficacy endpoints.
The committee’s role is to review safety and performance information and to provide recommendations on study continuation, suspension, termination, or protocol amendments as necessary.

The committee will be convened by the principal investigator when any of the following conditions arise:

- Major amendments to the study protocol.
- Occurrence of serious adverse events.
- Identification of significant issues during monitoring or auditing.
- Any other situation that the principal investigator considers requires review by the committee.

The results of the committee’s evaluations will be reported to the principal investigator, the institutional director, and the ethics review board.
Both the principal investigator and the institutional director will take appropriate action based on the committee’s recommendations.

**10. Benefits and Disadvantages of Participation**

**10.1 Potential Risks**

Both treatment regimens—scheduled intravenous acetaminophen and TEA—are established clinical practices used for perioperative pain control after gastrectomy.
Participation is not expected to expose patients to additional risk beyond standard care.
Adverse reactions are well known and will be monitored throughout the perioperative period.
All treatments are provided within the national health insurance system; no extra financial burden is imposed.

**10.2 Potential Benefits**

Participation is not expected to provide direct therapeutic benefit to individual patients.
However, the knowledge gained may contribute to optimizing postoperative analgesic strategies and improving recovery and quality of care in future practice.

## 10.3 Comprehensive Risk Assessment and Measures to Minimize Burden and Risk

To minimize potential risks and burdens associated with adverse events, the inclusion criteria (Section 4.1), criteria for dose or schedule modification (Section 6.2), and management of concomitant and supportive therapy (Section 6.4) have been carefully designed.

Regular monitoring will be conducted throughout the study to ensure that all adverse events remain within the expected range. The data center and the Efficacy and Safety Evaluation Committee will continuously review safety data. In the event of any serious or unexpected adverse event, a detailed review and evaluation will be undertaken in accordance with relevant institutional and regulatory procedures, and appropriate corrective or preventive actions will be implemented promptly to ensure participant safety.

# **11. Reporting to the Institutional Director**

Reports to the institutional director will include the items listed below and will be submitted in accordance with the internal regulations of each participating institution.

**11.1 Reports from Investigators**

Investigators shall promptly report to the institutional director in the following circumstances:

- Any breach of confidentiality or incident that may compromise the rights, safety, or welfare of study participants.
- Any event or information that could impair the integrity, validity, or credibility of the study or its results.

**11.2 Reports from the Principal Investigator**

The principal investigator shall report to the institutional director in the following cases and, if necessary, consider suspension, termination, or amendment of the study protocol:

- Any event or information that could compromise the ethical or scientific validity of the study and affect its continuation.
- Any occurrence that may jeopardize the proper conduct or credibility of the study.
- Progress reports on study implementation.
- The occurrence of adverse events related to the study.
- The occurrence of serious adverse events in this interventional study.
- Completion or premature termination of the study, accompanied by a summary report of the results.
- Final publication of study findings.
- The status of management of human biological specimens or personal data collected in the study.

# **12. Definition of Endpoints**

## 12.1 Primary Endpoint

The primary endpoint is the proportion of patients with a Numerical Rating Scale (NRS) pain score ≥4 at rest 24 hours after surgery (ie, in the evening of POD1).

This time point represents the period when incisional pain is expected to be most intense. According to the NCCN Clinical Practice Guidelines in Oncology (Adult Cancer Pain), an NRS score of 4–6 corresponds to moderate pain; therefore, the proportion of patients reporting moderate or greater pain at this time was selected as the primary outcome measure [11].

## 12.2 Secondary Endpoints

Secondary endpoints include the following:

- The area under the NRS–time curve during the first 72 hours (AUC₇₂) and the time courses of NRS scores at rest and during coughing
- Number of rescue analgesic administrations through POD 3.
- Patient satisfaction assessed by a 7-point Likert scale during POD 1–7.
- Anesthetic and operative time.
- Indicators of postoperative recovery, including time to first flatus, defecation, ambulation, and urinary catheter removal.
- Length of postoperative hospital stay.
- Incidence of adverse events related to postoperative analgesic treatment (CTCAE v4.0).
- Incidence of postoperative complications (Clavien–Dindo grade ≥II).

# **13. Statistical Analysis**

## 13.1 Sample Size Determination

In our previous multicenter randomized controlled trial evaluating postoperative analgesia after gastrectomy, the proportion of patients with an NRS score ≥4 at rest 24 hours after laparoscopic gastrectomy was observed to be 31.0% (9 of 29) in the TEA-alone group, which corresponds to the standard regimen in this study (7). Assuming a similar rate in both groups, a non-inferiority margin of 20 percentage points is defined as clinically acceptable.

With a one-sided significance level (α) of 0.05 and a power of 80%, the required sample size is calculated as 64 patients per group to demonstrate the non-inferiority of scheduled intravenous acetaminophen compared with TEA. Allowing for approximately 10% of dropouts or ineligible cases, the target sample size is set at 70 patients per group (total n = 140).

No interim analysis is planned.

## 13.2 Analysis Populations

Efficacy analyses are conducted primarily in the modified intention-to-treat (mITT) population, defined as all randomized patients who undergo gastrectomy and receive the allocated intervention. Patients who meet predefined discontinuation criteria—such as intraoperative conversion to open surgery, failed epidural catheter placement, intraoperative diagnosis of cStage IV disease, withdrawal of consent, or other conditions that prevent continuation according to Section 6.3—are excluded from the mITT population. A per-protocol (PP) analysis, which includes only patients who fully adhere to the assigned intervention without major protocol deviations, is performed as a sensitivity analysis to assess robustness. Safety analyses include all patients who receive any component of the assigned intervention.

## 13.3 Interim Analysis

No interim analysis will be performed in this study.

## 13.4 Analysis Variables and Statistical Methods

For the primary analysis, the proportion of patients with an NRS pain score of 4 or higher at rest 24 hours after surgery will be compared between groups. The absolute risk difference (acetaminophen − TEA) and its two-sided 95% confidence interval (CI) will be estimated using the Wald method for binomial proportions. Non-inferiority will be concluded if the upper bound of the 95% CI is less than the prespecified non-inferiority margin of +20 percentage points. The corresponding one-sided p value for non-inferiority will also be reported. To account for potential confounding, a supportive multivariable logistic regression model adjusting for the prespecified stratification factors will be fitted. Adjusted risk differences will be derived from marginal predicted probabilities. For secondary analyses, the area under the NRS–time curve (AUC₇₂) during the first 72 hours after surgery will be calculated using the trapezoidal method, based on serial NRS scores at each measurement point. Other secondary outcomes, including rescue analgesic use, patient satisfaction, and functional recovery parameters, will be compared using the Student t test, Mann–Whitney U test, χ² test, or Fisher exact test, as appropriate to the data distribution. All tests will be two-sided, and a p value < .05 will be regarded as statistically significant, except for the one-sided non-inferiority test for the primary endpoint.

# **14. CRFs: Completion and Submission**

**14.1 Types and Submission Deadlines**

CRFs used in this study and their respective submission deadlines are as follows:

1. Progress record: To be submitted within 4 weeks after discontinuation or completion of protocol treatment.
2. End-of-treatment report: To be submitted within 4 weeks after discontinuation or completion of protocol treatment.

**14.2 Instructions for Completion**

CRFs shall be completed in accordance with the prescribed format using a black or blue ballpoint pen. All entries must be legible, accurate, and consistent with the source documents.

**14.3 Submission Method and Contact Information**

Completed CRFs shall be submitted by postal mail or via email to the study data center.

Data Center:
Gastrointestinal Cancer Conference Study Group, Takara-machi Division
Department of Gastrointestinal Surgery, Kanazawa University Hospital
Tel: +81-76-265-2362
E-mail: gicc@med.kanazawa-u.ac.jp

# **15. Monitoring**

**15.1 Monitoring Procedures**

A monitoring officer designated by the principal investigator will conduct monitoring activities in accordance with the predefined monitoring plan.
The purpose of monitoring is to ensure that the study is conducted safely, in compliance with the approved protocol, and that all data are accurately and reliably collected.

Monitoring will be performed centrally by the data center, based on the case report forms (CRFs) submitted from each participating site.
On-site monitoring will not be routinely conducted; however, a site visit may be performed if the principal investigator or study representative deems it necessary.

The following items will be reviewed during monitoring:

1. Patient registration status
2. Eligibility of enrolled patients
3. Baseline characteristics before treatment
4. Treatment status (ongoing or completed) and reasons for discontinuation
5. Protocol deviations
6. Serious adverse events
7. Adverse reactions and other adverse events
8. Overall survival
9. Treatment-related deaths
10. Other issues related to study progress or safety

# **16. Ethical Considerations**

## 16.1 Regulatory and Ethical Compliance

All investigators and study personnel will conduct the study in accordance with the Declaration of Helsinki and the Ethical Guidelines for Medical and Health Research Involving Human Subjects issued by the Ministry of Education, Culture, Sports, Science and Technology and the Ministry of Health, Labour and Welfare of Japan.

All members involved in the study must be familiar with these principles and comply with them throughout the research process.

## 16.2 Preparation and Revision of the Informed Consent Documents

The principal investigator will prepare the informed consent form and participant information sheet and revise them as necessary.

Any newly created or revised versions must be submitted to the institutional review board (IRB) for review and approved by the institutional director before use. These documents will include all information required under the Ethical Guidelines for Medical and Health Research Involving Human Subjects and must not contain any content that could unduly influence participant decision-making.

If new findings arise during the study that may affect participant consent—such as updated safety information or the emergence of new treatment options—the consent materials will be revised accordingly and reapproved by the IRB and the institutional director prior to implementation.

## 16.3 Informed Consent

The informed consent process will comply with the requirements specified in the Ethical Guidelines for Medical and Health Research Involving Human Subjects.

The patient information sheet will include all elements necessary for informed consent as defined by the guidelines. The written consent form will follow the standard format of Kanazawa University Hospital, and written informed consent will be obtained from all participants prior to enrollment.

Before consent is obtained, the attending physician or investigator will explain the purpose, procedures, risks, and benefits of the study to the patient, allow sufficient time for consideration, and confirm the patient’s understanding of the study content. If the patient agrees to participate, both the investigator and the participant will sign and date the consent form. The form will include the names and signatures of the physician who provided the explanation and the participant, as well as the dates of explanation and consent.

Two copies of the signed consent form will be made: one will be given to the participant, and the other will be retained by the principal investigator. The original document will be filed in the patient’s medical record or stored in a designated secure location within the institution.

# **17. Protection of Personal Information**

All study personnel will comply with all applicable laws and institutional regulations regarding the protection of personal information.

They shall make every effort to protect the privacy and confidentiality of all study participants and must not disclose any personally identifiable information obtained in the course of the study without legitimate justification.

This obligation will remain in effect even after an individual’s involvement in the study or employment has ended.

All study-related documents and materials will be managed using unique identification codes unrelated to personal information, to ensure anonymity and confidentiality of participants.

When the study results are published, no personally identifiable data will be included.

Excised tissue samples may be used for future research that cannot be specified at the time of consent; such use will be conducted only after providing additional information and obtaining separate consent from participants.

All biological samples will be stored for 10 years after surgery, and no materials or data will be shared with external institutions or third parties.

The data protection officer responsible for the management of personal information in this study is Prof. Sachio Fushida, Faculty of Medicine, Kanazawa University.

**18. Funding, Conflict of Interest, and Compensation**

**18.1 Funding Source and Financial Relationships**

This work was supported by Gastrointestinal Cancer Conference Study Group (GICC). The funder had no role in study design, data collection, analysis, interpretation, or writing. It is investigator-initiated and conducted independently without commercial involvement. All investigators declare potential conflicts of interest under Kanazawa University COI policies.

**18.2 Study-Related Costs**

All medications, devices, and procedures are covered by the national health insurance system in Japan.
Therefore, participants incur no additional costs through study participation.

**18.3 Compensation for Health-Related Injury**

If a participant experiences health-related injury associated with this study, appropriate medical treatment is provided under the national health insurance system.
Participants are eligible for compensation under the Pharmaceutical Adverse Drug Reaction Relief System as defined by Japanese law.
These procedures are explained during informed consent.

# **19. Protocol Deviations, Amendments, and Revisions**

## 19.1 Protocol Deviations or Modifications

Investigators must not deviate from or modify the approved study protocol without prior agreement from the coordinating investigator and prior approval from the institutional review board (IRB) and the institutional director.

In exceptional or emergency circumstances where prior approval cannot be obtained, deviations or modifications may be implemented to protect the safety of participants. In such cases, the principal or sub-investigator shall promptly report the details and reasons for the deviation, along with any proposed protocol amendments, to the coordinating investigator, the IRB, and the institutional director for review and approval.
All deviations from the protocol and their reasons must be documented.

## 19.2 Protocol Revisions

Before study initiation, the principal investigator shall submit the protocol to the institutional director and obtain approval from both the IRB and the institutional director.
If protocol amendments become necessary, the principal investigator will evaluate the appropriateness and potential impact of the changes—consulting the study statistician or other experts as needed—and obtain IRB and institutional approval prior to implementation.
After approval, the revised protocol will be distributed promptly to all co-investigators, the data center, and other relevant study personnel.

# **20. Study Completion and Early Termination**

## 20.1 Study Completion

Upon completion of the study at each participating institution, the principal investigator shall promptly submit a final report and a summary of study results to the hospital director.
For multicenter studies, the final report shall also be submitted to the coordinating investigator and the study administrative office.

## 20.2 Early Termination of the Study

The principal investigator will review the feasibility of continuing the study if any of the following conditions occur:

1. Discovery of critical information affecting the quality, safety, or efficacy of the investigational treatment.
2. Significant difficulty in patient recruitment that makes completion of the planned sample size impracticable.
3. Instructions from the institutional review board to modify the study protocol that cannot be reasonably accepted by the investigators.

**21. Management and Retention of Study Documents**

The principal investigator is responsible for the proper management and retention of all study-related documents, including application forms, approval and notification letters, copies of submissions and reports, the subject identification code list, case report forms, and any other records necessary to ensure data integrity and reliability.

Electronic data and laboratory or observation notes will be retained for 10 years, and other study-related documents will be retained for 5 years after the later of either (1) the date of study completion report submission or (2) the date of final publication of study results.

After the retention period, all materials will be securely disposed of with due consideration for the protection of personal information.

Medical records will be stored and disposed of in accordance with the regulations of each participating institution.

**22. Ownership and Publication of Study Results**

This study will be registered with the University Hospital Medical Information Network (UMIN) prior to initiation.

All study results and related intellectual property will belong to the study group as a whole.　The coordinating investigator (principal investigator) and the statistician will jointly determine authorship based on the level of contribution, and the study findings will be presented at scientific meetings and published in peer-reviewed journals.

**23. Protocol Amendments Summary**

| **No** | **Date** | **Summary of Changes** | **Rationale / Comments** |
| --- | --- | --- | --- |
| 1 | 2020-02-26 | Original protocol approved by IRB. | Initial protocol registration and ethical approval. |
| 2 | 2021-09-24 | Change of study group representative. | Administrative update following staff reassignment; no change to study design or procedures. |
| 3 | 2021-12-28 | Eligibility criterion revised from “laparoscopic” to “laparoscopic or robot-assisted” gastrectomy to explicitly include both approaches. | Robot-assisted gastrectomy was initially considered part of laparoscopic surgery. As robotic procedures became common, the term was clarified. No impact on study design or population. |
| 3 | 2021-12-28 | Study group name changed from “DDSO” to “GICC.” | Administrative change due to leadership transition and reorganization; framework and protocol unchanged. |
| 4 | 2022-3-8 | Additional participating institutions added. | New centers joined under the same protocol with individual IRB approval to enhance patient accrual. Study design and endpoints unchanged. |
| 5 | 2023-1-11 | Co-investigators replaced or added due to personnel transfers. | Administrative update to investigator list and affiliations; no effect on study procedures. |
| 6 | 2023-02-02 | Study period extended by two years. | Extension approved owing to slower-than-expected enrollment; no change to design, endpoints, or analysis plan. |

# **24. participating trial centers**

| City | Name of Investigator | Affiliation |
| --- | --- | --- |
| Kanazawa | Noriyuki Inaki  Sachio Fushida  Jun Kinoshita  Hideki Moriyama  Toshikatsu Tsuji | Kanazawa University Hospital |
| Kanazawa | Shinichi Kadoya  Takahisa Yamaguchi | Ishikawa Prefectural Central Hospital, |
| Kanazawa | Shingo Soga | Kanazawa City Hopsital |
| Kanazawa | Yasumichi Yagi | Kanazawa Medical Center |
| Hakusan | Katsunobu Oyama  Yuki Yamazaki | Public Central Hospital of Matto Ishikawa |
| Toyama | Hideki Kaji  Tomoya Tsukada | Toyama Prefectural Central Hospital |
| Toyama | Takashi Fujimura  Shozo Sasaki | Toyama City Hospital |
| Toyama | Kazushige Shibahara | Toyama red cross hospital |
| Takaoka | Takuo Hara | JA Toyama Kouseiren Takaoka Hospital |
| Nanao | Toru Kamata | Keiju Medical Center |
| Fukui | Masanari Shimada | Fukui-ken Saiseikai Hospital |
| Yokohama | Toru Watanabe  Masahiko Kawaguchi | Yokohama Sakae Kyosai Hospital |

# **25. References**

1.Moraca RJ, Sheldon DG, Thirlby RC. The role of epidural anesthesia and analgesia in surgical practice. Annals of surgery. 2003;238(5):663-73. Epub 2003/10/28. doi: 10.1097/01.sla.0000094300.36689.ad. PubMed PMID: 14578727; PubMed Central PMCID: PMCPMC1356143.

2. Hermanides J, Hollmann MW, Stevens MF, Lirk P. Failed epidural: causes and management. British journal of anaesthesia. 2012;109(2):144-54. Epub 2012/06/28. doi: 10.1093/bja/aes214. PubMed PMID: 22735301.

3. Hughes MJ, Ventham NT, McNally S, Harrison E, Wigmore S. Analgesia after open abdominal surgery in the setting of enhanced recovery surgery: a systematic review and meta-analysis. JAMA surgery. 2014;149(12):1224-30. Epub 2014/10/16. doi: 10.1001/jamasurg.2014.210. PubMed PMID: 25317633.

4. Ljungqvist O, Scott M, Fearon KC. Enhanced Recovery After Surgery: A Review. JAMA surgery. 2017;152(3):292-8. Epub 2017/01/18. doi: 10.1001/jamasurg.2016.4952. PubMed PMID: 28097305.

5. Christian C Al, Alparslan T, Kimberly S, Joseph P, Cyrill H. Intravenous acetaminophen reduces postoperative nausea and vomiting: a systematic review and meta-analysis. Pain. 2013 May;154(5):677-689. doi: 10.1016/j.pain.2012.12.025

6. Mortensen K, Nilsson M, Slim K, Schäfer M, Mariette C, Braga M, et al. Consensus guidelines for enhanced recovery after gastrectomy: Enhanced Recovery After Surgery (ERAS®) Society recommendations. The British journal of surgery. 2014;101(10):1209-29. Epub 2014/07/23. doi: 10.1002/bjs.9582. PubMed PMID: 25047143.

7. Kinoshita J, Fushida S, Kaji M, Oyama K, Fujimoto D, Hirono Y, et al. A randomized controlled trial of postoperative intravenous acetaminophen plus thoracic epidural analgesia vs. thoracic epidural analgesia alone after gastrectomy for gastric cancer. Gastric cancer. 2019;22(2):392-402. Epub 2018/08/09. doi: 10.1007/s10120-018-0863-5. PubMed PMID: 30088162; PubMed Central PMCID: PMCPMC6394709.

8. Cho JS, Kim HI, Lee KY, Son T, Bai SJ, Choi H, et al. Comparison of the effects of patient-controlled epidural and intravenous analgesia on postoperative bowel function after laparoscopic gastrectomy: a prospective randomized study. Surgical endoscopy. 2017;31(11):4688-96. Epub 2017/04/09. doi: 10.1007/s00464-017-5537-6. PubMed PMID: 28389801.

9. Jota M, Shuji T, Yasuhiro M, Tsuyoshi T, Yukinori K, Makoto Y, et al. Novel management of postoperative pain using only oral analgesics after LADG. Surg Today. 2016 Jan;46(1):117-122. doi: 10.1007/s00595-015-1155-x. Epub 2015 Mar 24.

10. Japanese classification of gastric carcinoma: 3rd English edition. Gastric cancer : official journal of the International Gastric Cancer Association and the Japanese Gastric Cancer Association. 2011;14(2):101-12. Epub 2011/05/17. doi: 10.1007/s10120-011-0041-5. PubMed PMID: 21573743.

11. Swarm RA, Paice JA, Anghelescu DL, Are M, Bruce JY, Buga S, et al. Adult Cancer Pain, Version 3.2019, NCCN Clinical Practice Guidelines in Oncology. Journal of the National Comprehensive Cancer Network : JNCCN. 2019;17(8):977-1007. Epub 2019/08/08. doi: 10.6004/jnccn.2019.0038. PubMed PMID: 31390582.

12. Porembka MR, Hall BL, Hirbe M, Strasberg SM. Quantitative weighting of postoperative complications based on the accordion severity grading system: demonstration of potential impact using the american college of surgeons national surgical quality improvement program. Journal of the American College of Surgeons. 2010;210(3):286-98.
