# Supplementary material for: Scheduled intravenous acetaminophen versus thoracic epidural analgesia for postoperative pain control after minimally invasive gastrectomy: multicentre randomized non-inferiority trial
Source: BJS Open. 2026 Apr 27;10(2):zrag031. doi: 10.1093/bjsopen/zrag031 (PMC13111924; doi:10.1093/bjsopen/zrag031)
Supplement: zrag031_Supplementary_Data [file zrag031_supplementary_data.zip › Supplementary_material.docx]

**Title:**
Scheduled intravenous acetaminophen versus thoracic epidural analgesia for postoperative pain control after minimally invasive gastrectomy: a multicenter randomized non-inferiority trial

**Authors:**
Jun Kinoshita^1^, Saki Hayashi^1^, Toshikatsu Tsuji^1^, Hideki Moriyama^1^, Yuki Yamazaki^2^, Yuto Kitano^7^, Tomoya Tsukada^3^, Masahide Kaji^3^, Takahisa Yamaguchi^4^, Shinichi Kadoya^4^,　Yasumichi Yagi^5^, Masanari Shimada^6^, Shingo Soga^8^, Sachio Fushida^1,9^, Takumi Taniguchi^10^ and Noriyuki Inaki^1^, on behalf of the Gastrointestinal Cancer Conference study group.

**Affiliations:**
^1^Department of Gastrointestinal Surgery, Kanazawa University Hospital, Kanazawa, Japan

^2^ Department of Surgery, Public Central Hospital of Matto Ishikawa, Hakusan, Japan

^3^ Department of Surgery, Toyama Prefectural Central Hospital, Toyama, Japan

^4^ Department of Surgery, Ishikawa Prefectural Central Hospital, Kanazawa, Japan

^5^ Department of Surgery, Kanazawa Medical Center, Kanazawa, Japan

^6^ Department of Surgery, Fukui-ken Saiseikai Hospital, Fukui, Japan

^7^ Department of Surgery, Toyama Red Cross Hospital, Toyama, Japan

^8^ Department of Surgery, JCHO Kanazawa Hospital, Kanazawa, Japan

^9^ Non-Profit Organization (NPO) Digestive Disease Support Organization Study Group

^10^Department of Anesthesiology and Intensive Care Medicine, Kanazawa University Hospital, Kanazawa, Japan

**Corresponding author:**
Jun Kinoshita, MD, PhD
Department of Gastrointestinal Surgery, Kanazawa University Hospital
13-1 Takara-machi, Kanazawa 920-8641, Japan

ORCID ID: https://orcid.org/0000-0002-2871-9549

Tel +81 76 265 2362

Email junkino@staff.kanazawa-u.ac.jp

**Supplementary Materials - Index**

| **Supplementary Methods** |  |  |
| --- | --- | --- |
| Supplementary Table S1 | Institution-specific TEA regimens and patient allocation by treatment group | *pag. 2* |
| Supplementary methods | Statistical analysis | *pag. 3* |
| **Supplementary Results** |  |  |
| Supplementary Figure S1 | Exploratory post hoc GLMM analysis | *pag. 4* |
| Supplementary Table S2 | Pain-related secondary and exploratory outcomes in the per-protocol population | *pag. 5* |

**Supplementary Methods**

**Supplementary Table S1. Institution-specific TEA regimens and patient allocation by treatment group**

| **Institution** | **Local anesthetic** | **Opioid additive** | **Basal rate (mL/h)** | **Bolus (mL)** | **Lockout (min)** | **No. of patients (TEA / Acetaminophen)** |
| --- | --- | --- | --- | --- | --- | --- |
| A | 0.2% Ropivacaine 280–290 mL | Fentanyl 500–1000 µg | 4 | 3 | 60 | 32 / 30 |
| B | 0.2% Ropivacaine 300 mL | Morphine hydrochloride 2000–2500 µg/day | 4 | 3 | 60 | 18 / 18 |
| C | 0.2% Ropivacaine 280 mL | Fentanyl 1000 µg | 4 | 3 | 15 | 10/ 10 |
| D | 0.2% Ropivacaine 300 mL | Fentanyl 600 µg | 4 | 3 | 30 | 3 / 3 |
| E | 0.2% Ropivacaine 280 mL | Fentanyl 1000 µg | 4 | 3 | 30 | 3 / 3 |
| F | 0.2% Ropivacaine 200mL | Fentanyl 800 µg | 3 | None | None | 2 / 2 |
| G | 0.2% Ropivacaine 280 mL | Fentanyl 1000 µg | 4 | 3 | 30 | 0/1 |

**Abbreviations:** TEA: thoracic epidural analgesia

**Supplementary Methods: Statistical analysis**

**Secondary pain-related outcomes**

Secondary pain-related outcomes included the area under the NRS–time curve during the first 72 hours (AUC₇₂) at rest and during coughing, the number of rescue analgesic doses administered through postoperative day 3, and patient satisfaction. These outcomes were analyzed using the Mann–Whitney U test. Between-group effect estimates were expressed as Hodges–Lehmann median differences with corresponding 95% confidence intervals.

**Perioperative, recovery, and safety outcomes**

Perioperative and recovery outcomes included anesthetic and operative time, time to first flatus, time to first defecation, time to ambulation, time to urinary catheter removal, and length of postoperative hospital stay. Continuous variables were analyzed using the Student t test or the Mann–Whitney U test, depending on data distribution. Categorical outcomes, including surgical complications and analgesia-related adverse events, and laboratory toxicities were compared using the χ² test or Fisher’s exact test, as appropriate. Between-group differences and corresponding 95% confidence intervals were reported to facilitate descriptive comparison.

**Exploratory GLMM analyses of repeated binary pain outcomes**

Exploratory post hoc analyses of repeated binary pain outcomes, defined as an NRS score ≥4, were conducted using logistic generalized linear mixed models (GLMMs). The models included fixed effects for treatment group, postoperative time point, and their interaction, with adjustment for participating institution, type of gastrectomy, and surgical approach. A random intercept for patient was included to account for within-patient correlation across repeated postoperative assessments.

Adjusted risk differences (RDs) between treatment groups at each postoperative time point were calculated from the fitted models. Population-averaged predicted probabilities were obtained from the fixed-effects component of the models (re.form = NA) and averaged over the observed covariate distribution using marginal standardization. Adjusted RDs were defined as the differences between these standardized predicted probabilities.

95% confidence intervals were estimated using parametric bootstrap resampling. For each bootstrap replicate, outcome data were simulated from the fitted model, the model was refitted, and adjusted RDs were recalculated for each time point. Confidence intervals were defined by the 2.5th and 97.5th percentiles of the bootstrap distributions.

To account for multiplicity across exploratory postoperative time points (48 and 72 hours at rest, and 24, 48, and 72 hours during coughing), p values were adjusted using the Holm procedure. Confidence intervals were not adjusted for multiplicity.

**Supplementary Results**

**Supplementary Figure S1**

**
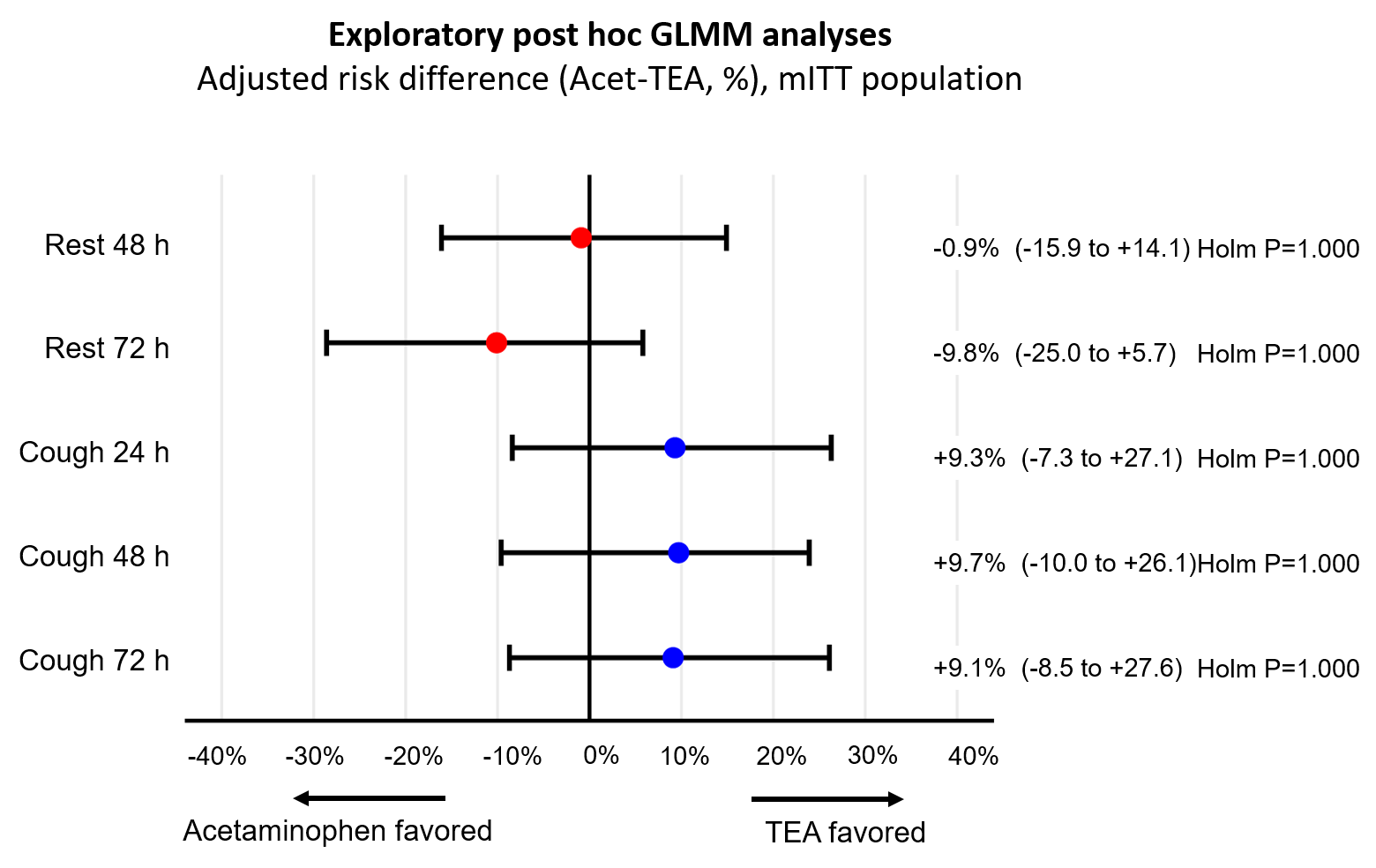
**

**Figure S1. Post hoc generalized linear mixed model (GLMM) analyses of pain outcomes.**Adjusted risk differences for inadequate pain (NRS ≥4) at postoperative assessment time points. Adjusted risk differences (acetaminophen − TEA) and 95% confidence intervals were estimated using GLMMs with fixed effects for treatment group, time point, and their interaction, adjusting for participating institution, type of gastrectomy, and surgical approach, and including a random intercept for patient. Adjusted risk differences were derived from population-averaged marginal predicted probabilities. Confidence intervals were estimated using parametric bootstrap resampling (percentile method). P values were adjusted using Holm’s procedure across five postoperative assessment time points (48 and 72 h at rest, and 24, 48, and 72 h during coughing); confidence intervals were not adjusted for multiplicity.

**Abbreviations:** Acet, acetaminophen; TEA, thoracic epidural analgesia.

**Supplementary Table S2. Pain-related secondary and exploratory outcomes in the per-protocol population**

**(a) Per-protocol sensitivity analysis of pain-related secondary outcomes**

| **Outcome** | **TEA group (n = 65)** | **Acetaminophen group (n = 67)** | **Effect estimate (95% CI)** | **p-value** |
| --- | --- | --- | --- | --- |
| AUC₇₂ at rest (median [IQR]) | 152 [72–252] | 140 [98–204] | +4.0 (−32.0 to +44.0) | 0.759 |
| AUC₇₂ during coughing (median [IQR]) | 300 [192–448] | 348 [250–444] | +28.0 (−20.0 to +80.0) | 0.255 |
| Number of rescue doses through POD3 (median [IQR]) | 1 [0–4] | 2 [0–3] | +0.0 (0.0 to +1.0) | 0.742 |
| Additional opioid rescue required, any (n [%]) | 0 (0 %) | 0 (0 %) | — | — |

**(b) Exploratory post hoc analysis-GLMM-adjusted risk differences for NRS ≥ 4 at rest and during coughing**

| **Outcome** | **Adjusted RD (95 % CI), % pt (Acet − TEA)** | **Holm P** |
| --- | --- | --- |
| At rest, 48 h | −2.0% (−15.7 to +11.4) | 1.000 |
| At rest, 72 h | −11.2% (−25.9 to +2.4) | 1.000 |
| During coughing, 24 h | +10.7% (-7.1 to +29.3) | 0.450 |
| During coughing, 48 h | +11.4% (−9.8 to +26.4) | 1.000 |
| During coughing, 72 h | +10.7% (−8.0 to +27.8) | 1.000 |

**Notes:**

(a) Values are presented as n/N (%) or median [IQR]. Effect estimates are presented as Hodges–Lehmann median differences (95% CI); p values were obtained from the Mann–Whitney U test.

(b) Adjusted risk differences were estimated using GLMMs with a random intercept for patient and fixed effects for treatment group, postoperative time point, and their interaction, with adjustment for participating institution, type of gastrectomy, and surgical approach. Adjusted risk differences were derived from population-averaged marginal predicted probabilities. P values were adjusted using Holm’s procedure across the five postoperative assessment time points; confidence intervals were not adjusted for multiplicity.

**Abbreviations:** NRS, numerical rating scale; GLMM, generalized linear mixed model; RD, risk difference; AUC₇₂, area under the curve over the first 72 hours; IQR, interquartile range; CI, confidence interval; POD, postoperative day; TEA, thoracic epidural analgesia.
